# Non-Enzymatic Antioxidants against Alzheimer’s Disease: Prevention, Diagnosis and Therapy

**DOI:** 10.3390/antiox12010180

**Published:** 2023-01-12

**Authors:** Angelica Varesi, Lucrezia Irene Maria Campagnoli, Adelaide Carrara, Ilaria Pola, Elena Floris, Giovanni Ricevuti, Salvatore Chirumbolo, Alessia Pascale

**Affiliations:** 1 Department of Biology and Biotechnology, University of Pavia, 27100 Pavia, Italy; 2 Department of Drug Sciences, Section of Pharmacology, University of Pavia, 27100 Pavia, Italy; 3 Department of Internal Medicine and Therapeutics, University of Pavia, 27100 Pavia, Italy; 4 Department of Drug Sciences, University of Pavia, 27100 Pavia, Italy; 5 Department of Neurosciences, Biomedicine and Movement Sciences, University of Verona, 37129 Verona, Italy

**Keywords:** Alzheimer’s disease, oxidative stress, antioxidants, flavonoids, vitamins, prevention, diagnosis, treatment, minerals

## Abstract

Alzheimer’s disease (AD) is a neurodegenerative disorder characterized by progressive memory loss and cognitive decline. Although substantial research has been conducted to elucidate the complex pathophysiology of AD, the therapeutic approach still has limited efficacy in clinical practice. Oxidative stress (OS) has been established as an early driver of several age-related diseases, including neurodegeneration. In AD, increased levels of reactive oxygen species mediate neuronal lipid, protein, and nucleic acid peroxidation, mitochondrial dysfunction, synaptic damage, and inflammation. Thus, the identification of novel antioxidant molecules capable of detecting, preventing, and counteracting AD onset and progression is of the utmost importance. However, although several studies have been published, comprehensive and up-to-date overviews of the principal anti-AD agents harboring antioxidant properties remain scarce. In this narrative review, we summarize the role of vitamins, minerals, flavonoids, non-flavonoids, mitochondria-targeting molecules, organosulfur compounds, and carotenoids as non-enzymatic antioxidants with AD diagnostic, preventative, and therapeutic potential, thereby offering insights into the relationship between OS and neurodegeneration.

## 1. Introduction

**Figure 1:**
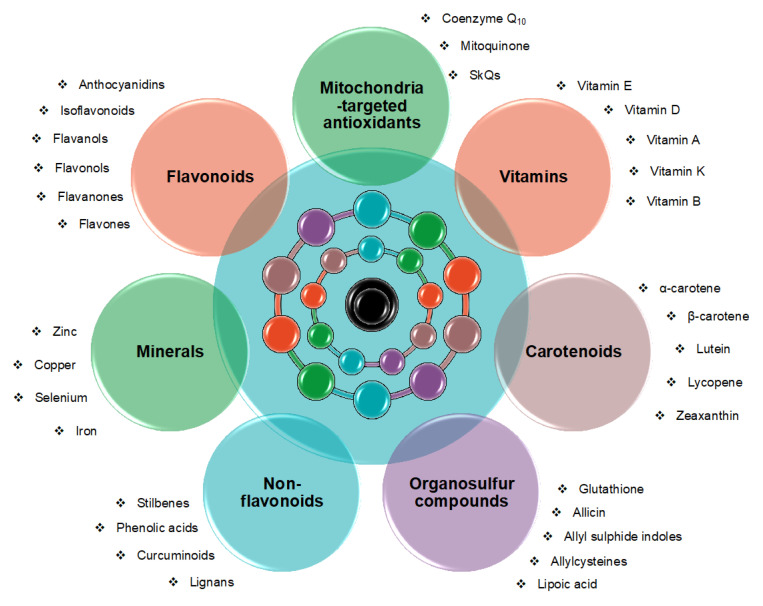
Antioxidant classification. The figure depicts the main classes of non-enzymatic antioxidants with potential diagnostic, preventative, and therapeutic applicability in the context of Alzheimer’s disease: mitochondria-targeted antioxidants, vitamins, carotenoids, organosulfur compounds, flavonoids, minerals, and non-flavonoids. The center depicts an iconic representation of a molecule with antioxidant properties.

Alzheimer’s disease (AD) is a progressive neurodegenerative disorder characterized by cognitive decline, memory loss, inability to complete simple tasks, and mood alterations [1]. To date, approximately 50 million AD cases have been registered worldwide, but this number is predicted to rise exponentially within the next few years [2]. Although several medications have been proposed, no disease-modifying therapy has been proven to be effective when tested in clinical trials [3]. Molecularly, AD presents a multifactorial etiology, in which genetic and environmental factors contribute to the formation of senile amyloid plaques, composed of amyloid beta (Aβ) fibrils, and intracellular neurofibrillary tangles (primarily characterized by the abnormal accumulation of the microtubule-associated tau protein, which is hyperphosphorylated) in different brain areas [4]. Among the risk factors, oxidative stress (OS) turns out to be one of the primary causes of AD, playing a key role in its pathophysiology and progression [5,6,7,8,9,10,11,12,13,14]. OS normally occurs because of an imbalance between pro-oxidant and antioxidant status, which leads to an excessive accumulation of reactive oxygen species (ROS), in turn causing oxidative damage at the level of biological macromolecules, such as lipids, proteins, and nucleic acids [15]. Concerning AD, there is evidence that an immoderate amount of ROS can increase Aβ fibril production and aggregation, tau phosphorylation, and neuronal cell death, as well as trigger a whole series of events leading to neurodegeneration (such as mitochondrial dysfunction and glial cell activation) [16,17]. In this context, it has been reported that oxidative damage to enzymes involved in glucose metabolism causes impaired adenosine triphosphate(ATP) biosynthesis and limits the brain’s energy availability in mild cognitive impairment (MCI) and AD patients [18,19]. At the same time, the decreased expression of the main antioxidant enzymes catalase (CAT), superoxide dismutase (SOD), glutathione peroxidase (GPx), and glutathione reductase reported in cognitively impaired individuals prevents the activity of the proper detoxification machinery [19,20,21]. This condition of oxidative imbalance, coupled with the consequent overexpression of nuclear factor kappa-light-chain-enhancer (NF-kB) and the release of various inflammatory mediators (i.e., interleukin 1 beta (IL-1β), IL-6, tumor necrosis factor alpha (TNF-α), and transforming growth factor beta (TGF-β)), concur to establish an age-related pro-inflammatory microenvironment that favors the onset of neurodegenerative disorders [21,22]. Once established, treatments aimed at curing the disease are often unsuccessful due to poor blood–brain barrier (BBB) penetration and the scarce bioavailability of the proposed drugs [21]. Thus, considering the harmful role of OS in AD, preventing the excessive formation of ROS may represent a useful potential strategy to counteract the onset and progression of this disorder, prior to its establishment. In this regard, the present review summarizes the preventative, diagnostic and therapeutic potential of several classes of non-enzymatic antioxidants, including carotenoids, vitamins, flavonoids, non-flavonoids, organosulfur compounds, mitochondria-targeted antioxidants, and minerals, with the aim of highlighting early biomarkers and promising preventative treatments for this devastating neurodegenerative disease (Figure 1).

## 2. Results

### 2.1. Carotenoids

Carotenoids are colorful pigments that are found in fruits, vegetables, and seaweeds, with important anti-inflammatory, antioxidant, and anti-apoptotic activity [23,24]. Chemically, carotenoids are composed of a polyene chain, enriched with conjugated double-carbon bonds, which are the basis of their redox potential [24]. Among the enormous variety of known carotenoids, α-carotene, β-carotene, lutein, zeaxanthin, lycopene and β-cryptoxanthin remain the most characterized and studied for their involvement in different pathologies and conditions [25,26]. Over the past few years, the discovery of their enrichment in certain brain areas [27] has led to the investigation of their diagnostic and therapeutic potential within the context of neurodegeneration [23,28]. Generally, low levels of blood carotenoids are detected among AD patients when compared to healthy controls [29,30], and plant-based diets are considered neuroprotective [31]. Among them, circulating levels of lutein, zeaxanthin, and lycopene are the most predictive of AD development, severity, and mortality among non-demented individuals, even when accounting for age, sex, genetic background, social status, and lifestyle, with a great potential for future application as prodromal AD biomarkers [31,32,33,34,35]. Moreover, since lutein and zeaxanthin are fundamental for retinal function, low serum levels of these carotenoids often accompany visual impairment and age-related macular degeneration (AMD) among AD patients [36]. Together with β-carotene, plasma lutein concentration is reported to distinguish severe AD patients from milder cases and healthy controls, thereby serving as a potential biomarker for patient stratification [37]. On the other hand, a higher circulating amount of the oxidized carotenoid, β-cryptoxanthin, is associated with better cognitive performance [38]. There is also evidence that circulating α-carotene and β-carotene may serve as non-invasive disease biomarkers, as they are significantly decreased in AD [39,40,41,42]. Accordingly, an analysis of 40 patients revealed that individuals presenting lower levels of plasma β-carotene showed increased concentrations of Aβ_1–42_ and total tau in the cerebrospinal fluid (CSF), thus linking peripheral β-carotene to established neurochemical AD markers [43]. Of note, plasma β-carotene correlation with telomerase activity may explain the association between AD development and aging [39]. However, inconsistent results and conflicting evidence are hindering clinical development. Indeed, neither differences in the circulating levels of α-carotene, β-carotene, lycopene, and β-cryptoxanthin between AD and controls, nor their association with dementia risk, were reported in other studies, thereby questioning the feasibility of these carotenoids for exploitation as disease biomarkers [34,35,40,44]. 

Besides diagnosis, a therapeutic supplementation of carotenoids has been proposed as an alternative curative as well as a preventative strategy for AD. 

In terms of **α-carotene** and **β-carotene**, it has been observed that streptozotocin-induced AD mice receiving β-carotene show better cognitive function and a reduced *A*β pathology, thanks to a diminished OS and the reduced activity of acetylcholinesterase (AChE), one of the most relevant proteins involved in AD [45]. However, a recent systematic review of the literature shows inconsistency regarding the use of β-carotene supplements to prevent MCI or AD in humans, thus requiring further investigation [46]. As for β-carotene, data from the “Modifying the Incidence of Delirium” (MIND) randomized controlled trial reported improved cognitive ability in individuals with higher levels of plasma α-carotene, although validation studies are still needed [47]. 

**Lycopene**. It has long been known that tomato consumption reduces the risk of developing cancer and cardiovascular disease, due to its high lycopene content [48]. Thanks to its antioxidant and neuroprotective functions, several studies proposed lycopene intake as a possible intervention against cognitive decline [48,49]. In this respect, preclinical evidence reported that lycopene supplementation is sufficient to improve learning and memory (evaluated via the Y-maze and Morris water maze tests) as well as to reduce Aβ accumulation and tau hyperphosphorylation in various mice and rat models of AD, even as a preventative strategy [50,51,52,53,54]. These benefits are mediated by decreased β-secretase expression, phosphatidylinositol 3-kinase (PI3K)/protein kinase B (Akt) signaling induction, and the stimulation of neurogenesis [52,53,55,56]. A reduction in OS, associated with decreased ROS production and enhanced antioxidant capacity (measured by nuclear factor erythroid 2-related factor 2 (Nrf2) activity, a reduced glutathione/oxidized glutathione (GSH/GSSG) ratio, malondialdehyde (MDA) levels, and GPx activity), was also observed across studies [52,54,55,56,57,58]. Often, lycopene-mediated restoration in oxidative homeostasis is accompanied by re-established mitochondrial morphology and functions [56,57,59]. All these events ensure cell viability and protect against apoptosis since in vitro lycopene treatment has been associated with reduced levels of cleaved caspase-3 and cytochrome c (markers for apoptosis), as well as with an increased expression of anti-apoptotic proteins at the expense of the pro-apoptotic proteins [55,56,59,60]. Moreover, the ability of neural stem cells to secrete nerve growth factor (NGF), brain-derived neurotrophic factor (BDNF), and vascular endothelial growth factor (VEGF) upon lycopene pre-treatment may further improve cell viability [61]. One of the key mechanisms of action of lycopene is its ability to counteract neuroinflammation. Indeed, AD rats receiving lycopene displayed reduced levels of serum pro-inflammatory cytokines (TNFα, IL-1β, IL-6β) and attenuated choroid plexus expression of the inflammatory mediators, toll-like receptor 4 (TLR4) and NF-kB, as well as increased CSF and hippocampal levels of the anti-inflammatory cytokines, IL-10 and TGF-β, even when treated at early disease stages [50,51,62]. Consistently, the same anti-inflammatory effects were observed upon the lycopene pre-treatment of mice subsequently injected with lipopolysaccharide (LPS) to trigger AD, suggesting that lycopene-based diets may be effective in disease prevention [52]. When tested in humans, results from an open-label interventional study involving 918 cognitively healthy subjects show that lycopene intake, combined with omega-3 fatty acids and *Ginkgo biloba* extracts, attenuated the risk of AD development later in life [63]. However, although some studies report an association between lycopene and cognitive function, human data remain limited [64]. Furthermore, innovative formulations based on lycopene-loaded microemulsions may also help to improve the antioxidant and neuroprotective properties of this carotenoid, as was recently shown in rats [65].

**Lutein, zeaxanthin, and meso-zeaxanthin**. AMD is often associated with AD [66], and macular pigment integration is fundamental to ensure both visual and cognitive function [67]. Accordingly, some studies show that lutein and zeaxanthin levels in the blood, macula, or brain are inversely correlated to MCI and AD occurrence, while their intake guarantees better cognitive reserve [68]. In vitro, the pretreatment of AD-mimicking neuronal cell lines with lutein and zeaxanthin extracts is reported to reduce neurotoxicity, limit apoptosis, prevent ROS release, and reestablish redox homeostasis [69,70]. In agreement with this concept, rats receiving zeaxanthin prior to Aβ_1–42_ exposure show reduced cerebrovascular inflammation, attenuated OS, and mitigation of the AD-related alterations in Aβ-metabolism [71]. These benefits may, at least in part, be explained by the ability of zeaxanthin to attenuate endoplasmic reticulum stress and mitigate tau hyperphosphorylation through modulation of the glycogen synthase kinase 3 beta (GSK-3β) pathway [72]. The preventative and protective features associated with macular pigment intake have also been confirmed in clinical studies. For example, cognitively healthy subjects receiving a mixture of lutein, zeaxanthin, and its stereoisomer, meso-zeaxanthin, for one year display marked memory improvements, thus potentially reducing the risk of AD development later in life [73]. Even more strikingly, 59 individuals, aged between 18 and 25 years, who received macular carotenoids for 6 months showed improved memory functions (both composite and verbal), increased attention, and enhanced cognitive performance compared to controls [74]. These effects seem to be mediated by reduced blood IL-1β, as well as by increased serum BDNF and macular carotenoid concentrations, which resulted in systemic antioxidant defense [74]. Nevertheless, results from the “Age-Related Eye Disease Study 2” (AREDS2) randomized trial report no significant benefits of lutein and zeaxanthin supplementation in terms of cognitive performance among individuals with a high risk of AMD development [75]. In another randomized double-blind clinical trial, the treatment of 31 AD patients with macular xanthophylls was not sufficient to observe any cognitive improvement, despite increased levels of blood lutein and zeaxanthin that were associated with the intake of these antioxidants [76]. Again, no amelioration in serum lipid oxidation was observed in AD patients under supplementation with lutein, zeaxanthin, and meso-zeaxanthin [77]. However, increased beneficial effects of macular carotenoids were observed upon their intake together with fish oil and omega-3 fatty acids, suggesting a dietary synergism [78]. Accordingly, cognitively healthy subjects aged over 65 years, on a diet supplemented for 2 years with a combination of fish oil, vitamin E, and macular pigments, showed improved cognitive ability, measured by working memory test performance, and increased levels of tissue carotenoids, as well as systemic xanthophylls and omega-3 fatty acid concentrations [79]. New formulations may also help carotenoids to achieve a higher therapeutic impact. In this respect, cationic biopolymer nanoparticles, endowed with efficient BBB permeation and brain localization, have been proposed as a vehicle for the intranasal delivery of carotenoids, and constitute a novel strategy to promote in situ antioxidant activity [80]. These innovative and targeted approaches may be used in new clinical trials to better clarify the preventative role of macular pigments within the AD context. 

### 2.2. Vitamins

The role of vitamins in AD and cognitive disorders has been extensively reviewed in recent years, wherein their function is usually associated with the ability to obstruct the impact of OS on neuroinflammation and neurodegeneration [81,82,83,84,85,86]. AD and its cognitive decline have been associated mainly with deficiencies in vitamins A, D, K, and E [85], while the most recent debate deals with the role of vitamin E in AD pathogenesis and progression [87]. 

**Vitamin E** is represented by a family of eight homologs that are synthesized by plants, starting from homogentisic acid (a phenolic acid). The family includes four tocopherols and four tocotrienols, divided into the α, β, γ, and δ forms, based on the methyl substitution in the aromatic ring [88]. Alpha-tocopherol is the predominant form in tissues and is, currently, the most extensively studied form [89,90,91]. Recent studies on the mechanism of action of these molecules indicate that γ-tocopherol, δ-tocopherol and γ-tocotrienol, besides influencing the immune function and cell signaling, together with lowering cholesterol levels, have unique antioxidant and anti-inflammatory properties, which are superior to those of α-tocopherol in the prevention and treatment of chronic diseases [92,93]. These forms of vitamin E are all scavengers of reactive nitrogen species (RNS), in addition to ROS; they inhibit cyclooxygenase (COX) and 5-lipoxygenase (5-LOX), catalyze the biosynthesis of eicosanoids, and suppress proinflammatory signaling. For this reason, these forms were extensively studied within the oxidative metabolism via in vitro investigation for their anti-inflammatory effect and, also, for their efficacy in preclinical models and in interventional human clinical trials [94,95]. Many biological activities that are ascribed in the literature to vitamin E offer a theoretical basis for an understanding of its beneficial effects in the treatment of AD. In this regard, recent evidence reports that vitamin E is particularly suited to preventing AD development. A possible explanation may come from the observation that vitamin E deficiency, when caused, for example, by mutations in the α-tocopherol transfer protein A (TTPA) in familial ataxia syndrome, triggers neurological disorders [96], thus indicating that this vitamin is particularly crucial to ensure an antioxidant response in the brain [97]. Undoubtedly, the role of vitamin E is paramount in reducing Aβ accumulation in the central nervous system (CNS), as observed in TTPA knock-out (Ttpa-/-) mice [98,99,100,101]. 

As mentioned, increased OS and inflammation are among the possible mechanisms involved in the pathogenesis of AD. Within this context, vitamin E has been proven to have positive effects on cognitive function [102]. When administered to AD mice, vitamin E was correlated with a reduction in Aβ in the brains of young mice, but not in aged animals [103]. Other studies carried out in animal models have shown that supplementation with α-tocopherol attenuates alterations of the Aβ metabolism in aged animals and prevents deficits in memory function [104]. Similar effects were observed for a metabolite of α-tocopherol (α-tocopherol quinine), the oral administration of which improves learning in amyloid precursor protein/presenilin-1 (APP/PS1)-transgenic mice, leading to a reduction in the brain levels of Aβ oligomers and determining a decrease in oxidative stress and in the production of inflammatory mediators [105]. In agreement with these findings, α-tocopherol was able to counteract the formation of Aβ oligomers and the toxicity induced by Aβ itself, reducing the inflammatory processes, the generation of ROS, and the oxidation of lipids in cell cultures [105,106,107]. 

Vitamin E was also documented as playing a neuroprotective role through the modulation of specific cell-signaling pathways. Indeed, studies performed on rats have shown that, at the hippocampal level, the deprivation of vitamin E is associated with the expression of numerous genes related to the development and progression of AD. These genes have been identified as important regulators of hormonal metabolism, apoptosis, growth, neurotransmission, and Aβ metabolism [108]. Consistently, low levels of cerebral α-tocopherol have been observed to induce downregulation of the genes involved in myelination and synaptogenesis, neuronal vesicle transport, and glial functions [109]. 

In addition, vitamin E inhibits several enzymes involved in neuroinflammation and oxidative damage, which are typical features of AD [110,111]. Furthermore, vitamin E activates protein phosphatase 2 (a phosphatase that plays a significant role in tau protein homeostasis), which has been shown to be downregulated in the brain of AD patients [112]. Recently, vitamin E has also been reported to be able to decrease cholesterol levels by affecting the pathway of sterol-regulating proteins [113]. Notably, numerous studies on cell cultures have shown that a reduced cholesterol content is associated with a decrease in the production of Aβ, while an increase in cholesterol has the opposite effect [114,115]. Accordingly, a strong positive correlation between hypercholesterolemia and increased Aβ levels has also been observed in animal models [115,116] and it has been documented that these effects are due to the direct stimulation of β- and γ-secretase activity by cholesterol [117,118]. Furthermore, the high cellular levels of cholesterol stimulate the internalization of APP, leading to an over-production of the substrate for β- and γ-secretases [119].

Besides having an impact on Aβ production, vitamin E and its derivatives also play an important role in tau functioning. Indeed, the treatment of neuronal cultures with vitamin E prevents the Aβ-induced hyperphosphorylation of tau, although the data are still conflicting [120]. The effect of vitamin E on tau was also analyzed in vivo in different animal models, where it was found that tau-transgenic mice supplemented with α-tocopherol display a reduction in disease development and improved health [121].

Concerning humans, low plasma levels of vitamin E have been observed in both MCI and AD subjects [102,122,123,124], while high plasma concentrations and a diet enriched in vitamin E have been associated with a reduced risk of developing AD [41,125,126]. On the other hand, elevated concentrations of vitamin E in the brain of AD patients suggest a possible compensatory response to the damaging oxidative stress conditions found in the CNS of these subjects [81]. Therefore, numerous studies have analyzed the effect of vitamin E supplementation on the progression of AD; however, they reached conflicting results. Actually, although some investigations have observed a slowing down in the progression of AD in patients treated with α-tocopherol [127], other authors have observed that vitamin E supplementation does not show any benefit in either MCI or AD patients [128,129]. 

Another vitamin that plays a key role in the brain is **vitamin A** (i.e., retinol, retinoic acid, and retinal and β-carotene); a deficiency in vitamin A is considered to be a fundamental biomarker of cognitive disorders [130]. Interestingly, vitamin A has been reported to be able to counteract the formation of Aβ in the AD brain [131,132,133,134]. Other vitamins, such as **vitamin K**, play a significant role in AD prevention and therapy [135]. For instance, low cerebral levels of vitamin K have been associated with worse cognitive decline and with the development of neurodegenerative diseases [136,137]. A deficiency in fundamental vitamins may also have a significant action on mitochondria [138,139]. A deficit in **vitamin D**, for example, impairs many of the functions associated with mitochondrial biology [140]. As a matter of fact, the depletion of vitamin D and the vitamin D receptor is fundamental for mitochondria activity [138,139], and this can surely help to explain why vitamin D is fundamental to preventing cognitive disorders, such as those occurring in AD [141,142].

The **B family of vitamins** was recently put in the spotlight because serum homocysteine is considered a potential risk factor for neurodegenerative disorders and the B vitamins, in particular vitamin B6 and vitamin B12, besides folic acid, are able to lower the serum levels of homocysteine, thus contributing to counteracting cognitive impairment [84,143,144,145,146]. By contrast, Li and colleagues reported no significant results in a meta-analysis about the effect of B vitamins on AD [147], whereas the findings of a previous meta-analysis, comprising 21 randomized controlled trials and 7571 participants, supported the intake of vitamin B to prevent cognitive impairment in adults [148]. Premature aging and neurodegeneration are the fundamental hallmarks of AD, which can be addressed by powerful antioxidant molecules, such as L-ascorbate [149]. A quantitative meta-analysis on the use of **vitamin C** in AD, performed on 12 studies of 1100 patients, concluded that a deficiency in vitamin C uptake is involved in the progression of AD [150], wherein a deficit in vitamin C is also associated with non-cognitive modifications, including anhedonia, decreased motivation, and sleep disorders due to a reduction of dopamine in the nucleus accumbens [151]. 

Fundamentally, vitamins in AD are able to reduce the impact of ROS and RNS as ignition factors for the mitochondria-released inflammasome, NRLP3, and the induction of neuroinflammation, acting either as antioxidant complexes or cofactors in the scavenger enzymes, thereby turning the debate on the crucial role of proper dietary habits in the elderly and during the course of one’s life [152].

### 2.3. Flavonoids

Flavonoids, a class of polyphenolic compounds produced by plants, are abundant in fruits and vegetables, as well as in tea and wine [153]. These compounds possess many biological properties, including antioxidant, anti-inflammatory, and anticarcinogenic activities [154,155], as a result of which they can potentially prevent neurodegenerative diseases [156,157]. In this regard, the intake of food rich in flavonoids, such as chocolate, wine, and green tea, was seen to be efficacious in terms of cognitive function in elderly people [158,159]. However, flavonoids’ benefits for humans are dependent on their method of intake and bioavailability [160]; indeed, considering that, in vivo, their bioavailability is low and they do not reach the concentrations necessary for exerting their antioxidant activity [155,161], the neuroprotection that they provide could be linked to additional mechanisms, including their effect on mitochondria and apoptosis [155,162].

Chemically speaking, flavonoids are divided into six subclasses: flavanols, flavanones, flavones, flavonols, isoflavones, and anthocyanins [163]. 

Catechins, which belong to the **flavanol** family, are antioxidant molecules found in various kinds of fruits (apricots, black grapes, and strawberries), vegetables (beans), as well as in tea and red wine [160]; they are able to scavenge free radicals, chelate metal ions, and induce antioxidant enzyme activity [164]. Based on their structure, eight main catechins have been identified, including epigallocatechin gallate, which is abundant in grapes, the seeds of leguminous plants, and tea [160,165]. 

Concerning AD, some in vitro studies have reported the beneficial role of flavanols in alleviating its pathological signs. They are able to reduce the formation of Aβ senile plaques and neurofibrillary tangles, prevent neuronal apoptosis [166], inhibit AChE activity, and activate the PI3K/Akt pathway, resulting in the inhibition of tau hyperphosphorylation (p-tau) [167]. Preclinical studies have highlighted the positive effects of different flavanols. For instance, Oligonol^R^, a flavanol-rich extract from lychee fruit, may improve cognitive impairment [168], while icariin, a flavanol contained in the medicinal herb *Epimedium sagittatum*, may attenuate synaptic plasticity and cognitive deficits through the activation of the BDNF/tropomycin receptor kinase B (TrkB)/Akt pathway [169], and cocoa extract administration may reduce Aβ oligomer formation [170]. Nevertheless, until now, no clinical trials have been performed; therefore, studies involving these flavanols are mandatory to better understand their role in AD. One of the most promising flavanols for the development of AD therapies is **epigallocatechin gallate** (EGCG), the main component in green tea, with neuroprotective mechanisms of action that have been widely studied, both in vitro and in vivo [171]. In the context of AD, some experimental studies have reported its anti-amyloidogenic and anti-inflammatory properties. EGCG may inhibit Aβ production by increasing the levels of the α-secretase, ADAM10, which is involved in the non-amyloidogenic pathway of APP, and converting mature fibrils into nontoxic protein aggregates, thereby reducing the overexpression of Aβ_1–40_ and Aβ_1–42_ [172,173,174,175,176]. Furthermore, its ability to modulate tau hyperphosphorylation has also been demonstrated [176,177,178]. As an anti-inflammatory compound, it is able to attenuate microglia activation and neuroinflammation by inhibiting the TLR4/NF-κB inflammatory pathway and alleviate neurotoxicity by reducing neuronal loss [179,180]. All these beneficial effects are able to counteract cognitive impairments in AD mice [174,175,181]. Interestingly, the combination of EGCG with other compounds endowed with a similar action has already shown encouraging results. For instance, the administration of hyaluronic acid with EGCG and curcumin inhibited Aβ aggregation [182], while treatment with EGCG and ferulic acid improved cognitive impairment and decreased Aβ levels [183]. However, although studies in AD animal models have shown promising results, clinical trials are still limited. In this respect, a randomized, double-blind, controlled clinical trial is currently evaluating the combination of lifestyle intervention with EGCG intake [184]. The results obtained from this study will be crucial in designing further clinical trials, with the aim of clarifying the actual impact that EGCG can have on the prevention and course of AD in humans. 

**Flavanones** are mostly found in citrus fruit and, at lesser concentrations, in tomatoes and mint, while **flavones** are abundant in celery and parsley [160]. 

**Hesperedin** (Hes) is a lipophilic flavanone that is present in oranges and lemons and is able to cross the BBB and provide neuroprotection [185]. Its aglycone, hesperetin (HPT), is known to be more bioavailable [160,186]. Thanks to their properties, including their neuroprotective effect [187,188,189], Hes and HPT may be potential candidates to manage neurodegenerative diseases [190]. Therefore, many studies focused their attention on these two flavanones as promising therapeutic agents for AD. Indeed, some in vivo animal studies highlighted the beneficial role of Hes in AD hallmarks, resulting in an improvement in memory deficit and behavioral impairments [191,192]. This flavanone is also able to reduce APP, Aβ_1–42_, and p-tau levels, and attenuate AChE activity, as well as prevent neuronal loss [185,193,194,195,196]. Furthermore, it may also act as an antioxidant and anti-inflammatory agent [188,197]. In AD mice, Hes reduces ROS, lipid peroxidation, and protein carbonyl, while increasing heme oxygenase 1 (HO-1), SOD, CAT, and GPx activities via activation of the Akt/Nrf2 signaling pathway and the inhibition of the receptor for advanced glycation end-products (RAGE)/NF-κB signaling pathway. Since RAGE is also a receptor for Aβ and the activation of this pathway results in neuronal OS and neuroinflammation, its downregulation seems to engender neuroprotection [198]. In another AD mouse model, Hes inhibited the overexpression of inflammatory markers, including NF-kB, inducible nitric oxide synthase (iNOS), cyclooxygenase-2 (COX-2), and the glial fibrillary acidic protein (GFAP) [199]. Despite the encouraging results obtained following these animal studies, only a few clinical studies have tried to elucidate the role of Hes in human neurodegenerative diseases. In a study conducted on healthy older adults, the consumption of Hes-rich orange juice improved cognitive function [200]; however, another study suggested that citrus intake more than two times a week is associated with a minor risk of developing dementia [201]. Nevertheless, the mechanism of action of Hes in the human body is still unclear; therefore, further clinical trials are needed to clarify and confirm the efficacy of this flavanone in neuroprotection and AD treatment. 

**Naringenin** (NGN) is one of the most abundant flavanones in citrus fruits, also found in grapefruits and tomatoes. Due to its lipophilic structure, NGN can cross the BBB [202] and some in vitro studies have shown that it is endowed with a neuroprotective role. NGN may inhibit pro-inflammatory cytokine, iNOS, and COX-2 expression through adenosine monophosphate-activated protein kinase α (AMPKα)/protein kinase C δ (PKCδ) signaling pathway activation [203]. In the context of AD, this flavanone can induce autophagy by activating AMPK/unc-51-like kinase (ULK1) axis, which leads to Aβ_1–42_ degradation [202], resulting in alleviation of the neurotoxic effects of Aβ-protein [204]. Furthermore, the administration of NGN nanoemulsion in SH-SY5Y cells that were exposed to Aβ downregulated APP and the expression of beta-site APP cleaving enzyme 1 (BACE1, which is the major secretase involved in Aβ production), as well as tau phosphorylation [205]. To support the hypothesis of NGN protection against neurodegeneration, further preclinical studies, performed using AD animal models, evaluated its effects. In this regard, the administration of NGN significantly enhanced cognitive deficits by reducing Aβ production, tau-hyperphosphorylation, AChE activity, neuroinflammation, and OS in the brain [206,207,208,209]. A clinical trial also studied the safety and pharmacokinetics of NGN in healthy adults and reported that the ingestion of 150 to 900 mg is safe [210]. However, there is a lack of clinical trials regarding the efficacy of NGN in AD. Nevertheless, from the evidence obtained in animal studies, NGN seems a plausible candidate for the management of AD and is worthy of further investigation. 

**Nobiletin** (NOB) is a flavone extracted from citrus peels, such as mandarins, sweet oranges, and lemons. Its beneficial properties, including its antioxidant, anti-inflammatory, and antiapoptotic effects, as well as its potential role in neurodegenerative diseases, such as AD and Parkinson’s disease (PD), have already been reported in the literature [211]. In this regard, several studies have highlighted its ability to suppress the expression of IL-1β, TNFα, iNOS, and COX-2, inhibit microglia activation via the mitogen-activated protein kinase (MAPK) and NF-κB signaling pathways, and increase the activities of GPx and manganese-superoxide dismutase (MnSOD) [212,213,214]. Concerning AD, several preclinical studies suggest the effects of NOB on the pathological features of AD. For instance, NOB can attenuate hippocampal neuroinflammation by lowering the levels of inflammatory cytokines and the transcription factor NF-kB, OS, and lipid peroxidation, and by strengthening the defense mechanisms against astrogliosis-associated neuroinflammation [212,215,216,217]. Moreover, NOB may also reduce Aβ_1–40_ and Aβ_1–42_ levels, as well as tau phosphorylation, resulting in the improvement of memory deficits and learning abilities [212,213,218,219]. Given the encouraging results obtained in animal studies, some clinical trials have also been performed. A randomized, double-blind, placebo-controlled study investigated the benefits and safety of this flavone in the elderly, finding that the administration of NOB-containing food had a beneficial effect on memory in people with MCI, compared to the placebo group [220]. Nevertheless, this study was not conducted in AD patients. In this regard, one observational study performed in AD subjects showed no significant changes in cognitive functions in patients treated with donepezil (an acetylcholinesterase inhibitor that is commonly used for AD treatment) in association with a herbal medicine containing NOB-rich *Citrus reticulate*, compared to the group only treated with the drug [221]. Conversely, another study conducted on 6 patients reported the beneficial effect of Nchinpi, a traditional drug containing NOB, combined with donepezil in patients with mild-to-moderate AD. Indeed, after one year, the cognitive state worsened only in the control group, pointing out the potential effectiveness of this combination in preventing the progression of cognitive impairments in AD patients [222].

**Apigenin** (API) is a flavone found in various plants, including chamomile, Melissa officinalis, parsley, thyme, and oregano, in vegetables, such as onions and celery, and in citrus, offering anti-inflammatory, antioxidant, anti-amyloidogenic, and neuroprotective properties [223,224]. Thanks to these effects, API could be a potential candidate to prevent or slow down the progression of AD. Indeed, several in vitro and in vivo preclinical studies reported its ability to attenuate AD hallmarks. API may be endowed with a protective role against neuronal damage by reducing neuroinflammation through the inhibition of OS and the TLR4/NF-κB inflammatory pathway [225,226]. Moreover, it has been demonstrated that this flavone may reduce Aβ formation and tau hyperphosphorylation by decreasing the BACE1 and GSK-3β levels, respectively, thus favoring an improvement in memory and learning deficits [223,224,227,228]. Interestingly, vitexin, an API flavone glycoside, was seen to reduce Aβ peptide expression and increase the lifespan in *Caenorhabditis elegans* with AD [229]. To assess the effects of API in humans with AD, Balez et al. used a human induced pluripotent stem-cell model of AD, in which skin cells derived from both patients and healthy individuals were cultured and differentiated into neurons. They noticed that API administration reduced neuronal apoptosis and inhibited the activation of cytokines and nitric oxide (NO) production [230]. Overall, considering the substantial evidence for API’s neuroprotective efficacy, both in vitro and in vivo, further studies and clinical trials should be encouraged. 

The most widely studied **flavonols**, antioxidant molecules that are abundant in multiple fruits and vegetables, as well as in tea and red wine, are quercetin, fisetin, kaempferol, and myricetin [231]. 

**Quercetin** is an aglycone form of flavonoid glycosides extracted from grapes, onions, berries, broccoli, and citrus [232]. Several studies have indicated its protective role in different pathologies associated with dementia, such as stroke and cardiovascular disease, as well as in aging. In the context of AD, quercetin is found to improve memory deficits and learning functions in several rodent models [232,233,234,235,236]. Furthermore, the efficacy of the intake of quercetin-rich onions in improving memory in early-stage AD patients has been reported [237], although the study by Holland et al. showed no enhancement in cognitive activities after quercetin administration [238]. Quercetin also possesses anti-inflammatory, antioxidant, and anti-amyloidogenic properties [232,234,236,239]. Regarding this last point, some in vitro and preclinical studies have highlighted quercetin’s ability to inhibit Aβ aggregation [240,241] and reduce Aβ accumulation and plaque generation by decreasing the BACE1 protein levels [236]. Despite this evidence, a study by Huebbe et al. [242] reported no effects of quercetin on the mRNA levels of genes involved in AD, especially BACE1. As an anti-inflammatory agent, quercetin may modulate neuroinflammation by suppressing the expression of the ionized calcium-binding adapter molecule 1 (Iba-1) and GFAP, which are typical proteins of activated microglia and astrocytes, respectively. Furthermore, it was reported that quercetin inhibited the TLR4/NFKB pathway involved in inflammatory signaling, as well as multiple inflammatory mediators such as COX-2, NOS-2, IL-1β, prostaglandins, and leukotrienes [232,236]. In addition, as an antioxidant molecule, quercetin is capable of decreasing MDA levels and increasing SOD and glutathione (GSH) expression, as well as modulating the Nrf2 transcription factor, with the consequent gene expression of several anti-antioxidant enzymes [232,236]. Moreover, quercetin is able to reduce tau phosphorylation [234,235], modulate mitochondrial dysfunction by increasing the mitochondrial membrane potential and ATP synthesis, decreasing ROS expression; it also displays a neuroprotective antiapoptotic function [236,241]. Evidence has indicated that even though quercetin crosses the BBB, its permeability is poor and its concentration in the brain is low. This may be due to the fact that quercetin is a substrate for the BBB efflux transporter, P-glycoprotein [242]. Therefore, promising delivery systems, including quercetin lipid nanoparticles, have been investigated and have proven efficient as potential future pharmaceutical approaches [243,244,245].

Another important flavonol investigated as a valuable neuroprotective compound for AD is **fisetin**, which is not particularly abundant in fruits (strawberries, kiwi, peaches, and grapes) and vegetables (tomatoes and cucumbers) [246]. Concerning AD, several animal studies have reported the effectiveness of fisetin in alleviating AD signs. On a molecular level, the administration of this flavonol may decrease Aβ production and aggregation by reducing BACE1 expression, tau hyperphosphorylation [247,248], neuroinflammation, and GFAP [246,247]. Moreover, as an antioxidant agent, fisetin is able to counteract protein carbonylation and lipid peroxidation [246,249], as well as to favor Nrf2 activation, with a consequent increase in GSH levels [246,248]. Thanks to these beneficial properties, the ability of fisetin to counteract cognitive impairment [246,247,249] and locomotor deficits has been reported [250]. Nevertheless, given that dietary fisetin sources are inadequate for effectiveness in humans, advanced delivery systems and derived compounds are needed [246]. 

Interestingly, many current studies indicate the potential effect of other compounds derived from flavonols in AD treatment. An in vitro study by Jung et al. reported the role of sophoflavescenol, a prenylated flavonol extracted from *Sophora flavescens*, in inhibiting BACE1 and cholinesterase activities [251]. In addition, treatment with icaritin, another prenylated flavonol, improved cognitive dysfunction, and reduced amyloid deposition, as well as tau phosphorylation, in AD mice [252]. Finally, the study conducted by Calderon-Garciduenas et al. among urban children suggested the beneficial effects of cocoa and dark chocolate administration against OS and neuroinflammation [253]. However, some studies reported controversial results concerning the impact of flavonols on AD hallmarks. For instance, the treatment with *Ginkgo biloba* extracts did not show any effect on Aβ aggregation and BACE1 activity [254,255]. 

**Isoflavonoids** are compounds mainly found in soybeans and other leguminous plants. While daidzein, daidzin, genistin show benefits narrowly in hormone-related diseases, genistein has been widely studied as a treatment in neurodegenerative disorders [231]. 

Regarding this last compound, **genistein** (GNT), the main phytoestrogen of the *Leguminosae,* has several properties, including antioxidant, antimicrobial, antitumor, and neuroprotective effects [256,257,258], and could be a potential candidate for AD treatment [257]. In this regard, some in vitro studies have shown the ability of this isoflavonoid to reduce Aβ production and deposition, reduce APP phosphorylation, and inhibit the activity of AChE [258,259,260]. Nevertheless, a study on SHSY5Y cells reported an increase in both APP and β-secretase mRNA and protein expression, thus going against the concept of a positive role for GNT in AD [261]. Additionally, a study on an LPS mouse model showed reduced AChE activity in the hippocampus after GNT oral administration [262], while Yu-Xiang Wang et al. reported its neuroprotective effect against Aβ_25–35_, via the modulation of choline acetyltransferase (ChAT) expression [263]. In terms of AD, several in vitro and in vivo studies have shown the beneficial role of GNT administration in ameliorating the symptoms of the disease. For instance, GNT can inhibit APP expression, thus reducing Aβ production and its following aggregation, and modulate tau phosphorylation, with a consequent improvement in memory and learning abilities [258,259,260,264,265,266,267,268,269]. However, a recent clinical trial reported controversial results after soy isoflavone treatment; indeed, Gleason et al. found no cognitive changes in AD patients [270]. Besides these beneficial properties, GNT has also been proven to have antioxidant and anti-inflammatory effects. In this regard, GNT has been reported to be able to counteract Aβ-induced oxidative injury by decreasing ROS accumulation and MDA levels, as well as by increasing antioxidant enzyme activity (in SOD and GSH) [262,269], presumably through the activation of the PI3K/Akt/Nrf2 signaling cascade [271]. Moreover, as an anti-inflammatory agent, GNT seems to be involved in the inhibition of the pro-inflammatory cytokines, IL-6 and TNFα, and other inflammatory or inflammation-associated factors, such as NF-kB, COX-2, iNOS, and GFAP [262,272]. Since GNT has poor oral availability, with limited absorption and considerable metabolization by the gut microbiota [257], recent studies have explored the use of nanoconjugates, in order to increase GNT’s BBB crossing and better modulate brain distribution [273]. Finally, interesting studies have also focused on the use of GNT-O-alkylamine derivatives as a potential AD treatment, finding antioxidant and AChE-inhibitory effects [274,275]. 

**Anthocyanins** are a class of pigments that are extracted from plants, in particular, soybean seeds and berries [276]. The main dietary anthocyanins are cyanidin, delphinidin, malvidin, peonidin, pelargonidin, and petunidin [277]. These compounds have been shown to display multiple activities, including antioxidant, anti-inflammatory, and anti-apoptotic effects [278]. These properties, as well as their effective role in tau hyperphosphorylation and Aβ amyloidosis regulation, could make them potential candidates for AD treatment [278]. A clinical study by Shishtar et al. [279] reported the efficacy of long-term anthocyanin intake in reducing the risk of AD and other related dementias. In addition, an anthocyanin-enriched juice has been found to have beneficial effects in improving cognitive function in middle-aged women [280], indicating the potential role of anthocyanins, not only in AD-related dementia but also in the aging process [277]. The neuroprotective effects of anthocyanins have also been documented in AD mouse models, with consequent improvement in learning and memory abilities [281,282,283,284,285,286,287,288]. Some preclinical studies have also reported their anti-Aβ aggregation activity [289,290,291]; additionally, they are able to reduce the amount of Aβ_1–40_ and Aβ_1–42_ and alter the APP metabolism by increasing the cleavage toward the production of soluble amyloid precursor protein-α (sAPPα), a neurotrophic factor [276,277,292]. Furthermore, treatment with anthocyanins was demonstrated to significantly reverse and regulate BACE1 expression and protect against Aβ toxicity [276,286,292,293]. Regarding anthocyanin’s effects on tau-proteins, the results are controversial; some research carried out on AD mouse models reported a slight improvement in tau protein expression [286] and a reduction in p-tau hyperphosphorylation [292], while other studies showed unaltered levels of phosphorylated tau after anthocyanin administration [276]. These pigments also possess antioxidant and anti-inflammatory properties. Both the in vitro and in vivo studies demonstrated that anthocyanins are able to decrease lipid peroxidation and raise Nrf2 nuclear translocation, as well as raise the levels of antioxidant enzymes, including SOD, CAT, and GPx [277,289,294,295,296,297]. As anti-inflammatory agents, they may reverse microglia and astrocyte activation, resulting in the downregulation of the pro-inflammatory cytokines (TNFα, IL-1β, IL-6), COX-2, and iNOS [282,286]. Mechanistic studies have reported anthocyanins as molecules that are also able to suppress neuroinflammation, through the inhibition of NF-kB and Jun N-terminal kinase (JNK) activation [277,284]. Moreover, anthocyanins have been shown to modulate the mitochondrial apoptotic pathway by regulating different enzymes (caspases 3, 7, 9) and pro-apoptotic proteins in several AD mouse models [276,282,292,294]. Although anthocyanins are absorbed as glycosides at very low levels, they have been documented to cross the BBB in both rodents and humans, and exert sufficient biological activities, including gene regulation and cell signaling [276,277]. Within this context, nanodrug delivery systems have been developed for the purpose of achieving better absorption, bioavailability, and effectiveness. For instance, anophytosome formulations targeting the mitochondria, and polyethylene glycol–gold nanoparticles loaded with anthocyanins, have proven more effective than treatments using free anthocyanin [293,297,298]. 

Overall, flavonoids comprise a broad range of antioxidant compounds, with great translational potential as supportive AD treatments. The results from new, randomized trials involving AD and MCI patients should better elucidate the optimal time for treatment and offer insights into their possible use as preventative strategies for this devastating disease. 

### 2.4. Non-flavonoids

Another potential approach to preventing or treating AD could be the assumption of non-flavonoid substances, natural compounds that are mainly present in fruits, vegetables, green tea, and whole grains, with effective antioxidant properties [299,300]. The ability to attenuate OS and scavenge free radicals could make them possible candidates for counteracting neurodegeneration and improving the typical signs of neurodegenerative diseases [301]. In terms of AD, several studies reported the beneficial effects of non-flavonoids in managing this pathology (Table 1) [302,303,304,305,306,307,308,309,310,311,312,313,314,315,316,317,318,319,320,321,322,323]. 

Non-flavonoid compounds include phenolic acids, stilbenes (resveratrol), curcuminoids (curcumin), and lignans [299]. 

**Phenolic acids**, a class of secondary metabolites that are widely present in herbs and, more generally, in the plant kingdom, are divided into two groups, based on their chemical structure: hydroxycinnamic acids (including ferulic, caffeic, and p-coumaric acids) and hydroxybenzoic acids (including gallic and ellagic acids) [324,325]. In addition to possessing anti-inflammatory, antibacterial, anticarcinogenic, and neuroprotective properties [326,327], they are considered excellent as both direct and indirect antioxidants, able to scavenge excessive free radicals and activate the endogenous antioxidant pathways and enzymes, respectively [328]. 

**Ferulic acid** (FA), commonly abundant in oranges, tomatoes, spinach, grains, and wheat bran, and largely used in the cosmetics and food industries, is one of the most studied phenolic acids for its neuroprotective, antioxidant, and anti-inflammatory effects [329,330,331,332,333]. Due to its structure, FA is a strong free-radical scavenger; moreover, it also inhibits ROS formation by reducing pro-oxidant enzyme levels and promoting antioxidant enzyme activities [330]. 

The neuroprotective effect of this compound has been widely reported in many experimental studies on PD, cerebral ischemia/reperfusion injury, and multiple sclerosis (MS) [333,334,335,336]. In the context of AD, using in vitro and in vivo animal models, FA has been proven to have anti-amyloid, antioxidant, and anti-inflammatory effects, resulting in the improvement of cognitive impairment and the alleviation of neuropathological signs [337]. In this regard, FA can inhibit Aβ fibril aggregation and the resulting Aβ senile plaque formation by reducing the expression of BACE1 and APP levels [302,338,339,340]. It can also reduce tau levels and disassociate the pre-formed neurofibrillary tangles [337]. As antioxidant agents, FA and its derivatives may attenuate ROS formation by increasing SOD activity and reducing MDA levels [303,341,342,343]. In particular, butyl ferulate, a lipophilic FA derivative, has been shown to inhibit Aβ_1–42_ aggregation, reduce ROS accumulation, and increase antioxidant enzyme expression (such as HO-1, thioredoxin 1, and GSH) by activating the Nrf2 antioxidant signaling pathway, the most important cellular antioxidant defense cascade [344]. Furthermore, as anti-inflammatory compounds, they may alleviate neuroinflammation by reducing some of the pro-inflammatory enzymes (COX-2 and 5-LOX) and cytokines (TNFα, IL-1β, and IL-6) in the brains of AD mice [17,338,345]. In addition, given that, in the presence of neuronal damage, astrocytes and microglia overproduce GFAP and Iba1, two markers of astrogliosis and microglia activation, respectively, it has been reported that FA administration can reduce the levels of these proteins [183,342]. However, notwithstanding these beneficial properties, to date, no clinical trials have been performed to study the effects of FA on AD patients; therefore, future studies are mandatory to confirm these valuable results, obtained from both in vitro and in vivo models. 

**Caffeic acid** (CA), which is abundant in fruits (strawberries and blueberries), vegetables (carrots and tomatoes), and beverages (wine, tea, apple juice, and coffee) [346], has a broad spectrum of functions, including anti-inflammatory, antioxidant, anticancer, and immunomodulatory effects [347,348,349]. CA can work as an antioxidant and is able to prevent ROS production, scavenge free radicals, and chelate metals (such as ferrous ions) [350,351]. Besides these important activities, its neuroprotective role in cerebral ischemia/reperfusion injury, PD, brain lesions, and MS has been reported [352,353,354,355]. 

To date, few in vitro and in vivo studies have been performed to evaluate its anti-AD effects. In particular, a neuroprotective role of caffeic acid phenethyl ester (CAPE), abundant in the honeybee, and caffeic acid phenethyl ester 4-*O*-glucoside (FA-97), its synthetic derivative, has been demonstrated. Their administration may counteract OS and reduce ROS production by inducing the activation of the Nrf2/HO-1 pathway, resulting in increased SOD and GSH levels [306,356]. CA has also demonstrated the ability to inhibit AChE activity, restoring the levels of acetylcholine (ACh), a neurotransmitter with an important role in cognitive function [357,358]. This modulation alleviates cholinergic neuronal loss, improves cognitive functions, and attenuates learning and memory deficit in AD animal models [305,346,357]. Furthermore, it has been demonstrated that CA, with its anti-amyloidogenic activity, can exert a neuroprotective effect against β-amyloid-induced neurotoxicity [304]. 

Nevertheless, despite the encouraging results obtained from these studies, even for this compound, clinical trials are needed to better assess its potential therapeutic role in managing AD.

**p-Coumaric acid** (p-CA), a plant metabolite that is widely found in vegetables (carrots, tomatoes, garlic, and navy beans), fruits (apples, grapes, and oranges), peanuts, and cereals [359,360], is endowed with antioxidant, anti-inflammatory, antiangiogenic, and antitumor potential [361,362,363,364]. As an antioxidant agent, p-CA can reduce OS by activating antioxidant enzymes, and constitutes an effective free radical scavenger and metal chelator [365,366]. A powerful neuroprotective effect against embolic cerebral ischemia, cerebral ischemia-reperfusion injuries, and brain damage has also been reported [367,368,369]. 

Regarding its potential role in AD, few in vitro and preclinical studies have highlighted p-CA’s anti-amyloidogenic, antioxidant, and anti-inflammatory effects [307,308,370,371]. Indeed, this compound may reduce Aβ generation by inhibiting BACE1 activity, relieve ROS generation by increasing SOD and GSH expression, and modulate neuroinflammation via NF-kB pathway inactivation [307,308]. Since NF-kB plays an important role in the inflammatory response, its suppression causes the under-expression of two pro-inflammatory enzymes, iNOS, and COX-2, with a consequent attenuation in Aβ-induced toxicity [307]. Moreover, p-CA can also protect against learning and memory impairments and neuronal loss by reducing brain AChE activity [308]. Interestingly, it has been shown that maltolyl p-coumaric, a synthesized compound, is able to counteract cognitive deficits and exert neuroprotective effects in a rat model by reducing ROS and neuronal loss [309]. 

All this evidence suggests the potential therapeutic use of p-CA against AD; however, clinical studies are necessary to better elucidate its effects in humans and the molecular mechanisms underlying its possible pharmacological employment against AD. 

**Gallic acid** (GA) is a natural secondary metabolite that is abundant in the plant kingdom, especially in fruits (grapes, blackberries, strawberries, plums, and pomegranates) and nuts, as well as in tea and wine [372,373]. This compound and its derivatives are largely used in the food and pharmaceutical industries, thanks to their powerful properties, including their antioxidant, anti-inflammatory, anticancer, and antiviral effects [374,375,376,377,378,379]. Besides these activities, it has also been observed that GA plays a neuroprotective role in traumatic brain injury, PD, amyotrophic lateral sclerosis (ALS), and cerebral ischemia-reperfusion, as well as in psychiatric disorders [380,381,382,383]. 

Concerning AD, few in vitro and in vivo studies report the potential therapeutic role of GA in improving the neuropathological hallmarks of the disease. It has been documented that GA administration can stabilize OS and alleviate neuroinflammation, resulting in improved neurodegeneration by increasing antioxidant enzyme brain expression (CAT, GSH, SOD, GPx1, and SOD1) and decreasing reactive astrocytes and microglia cell activation in AD animal models. Moreover, its efficiency has been observed in reducing Aβ_1–42_ aggregation and amyloid plaque formation through β-secretase modulation [310,311,384]. In addition, this molecule was found to be capable of ameliorating cognitive impairments and preventing neuronal apoptosis in AD rats [310,385]. Interestingly, tannic acid, a glucoside polymer of GA, prevents the reduction of antioxidant enzyme activities, as well as an increase in AChE activity, in the proinflammatory cytokine, IL-6, and also TNFα levels, as seen in a model of sporadic dementia of the Alzheimer’s type (SDAT). Furthermore, this compound was also described as being able to favor neuronal survival by re-establishing the levels of Akt and pAkt that are involved in this process [386]. Although these studies have highlighted the neuroprotective effects of this phenolic acid and its derivatives in AD, clinical studies are needed to investigate their efficacy and safety in humans. 

**Ellagic acid** (EA), a natural polyphenol that is abundant in fruits (strawberries, raspberries, and pomegranates) and nuts, demonstrates antioxidant and anti-inflammatory properties that indicate a potential neuroprotective agent against several neurological disorders [387,388,389,390]. The antioxidant capacity is given by its ability to scavenge free radicals, chelate ions, inhibit lipid peroxidation, and promote antioxidant enzyme activity [391,392,393]; instead, as an anti-inflammatory compound, EA may reduce pro-inflammatory factor expression [393]. 

To date, few studies report EA’s potential effects in alleviating AD symptoms [312,313,314]. Using different in vivo AD animal models, its effectiveness has been demonstrated in improving learning and memory deficits, due to its ability to relieve OS (↑CAT, SOD, and GSH), neuroinflammation (↓NF-kB), AChE activity, and tau phosphorylation, this latter event occurring via the modulation of the Akt/GSK-3β signaling pathway [312,313]. Furthermore, this compound may also have an anti-amyloidogenic effect. Indeed, it is capable of decreasing Aβ deposition and plaque formation by reducing BACE1 activity and APP phosphorylation [312,314]. 

Despite these studies reporting the beneficial effects of EA as a potential AD treatment, more in vivo experimental studies, beyond all the clinical trials, are mandatory to confirm these findings. 

Among the stilbenes family, **resveratrol** (RSV) represents the most common polyphenolic compound, which is mainly found in grapes, mulberries, and peanuts, as well as in wine and grape juice [394,395]. It is well known for its ability to act as a strong antioxidant and anti-inflammatory agent, making it effective against different neurological diseases, including epilepsy, PD, Huntington’s disease, ALS, and neuronal injury [395]. RSV is able to scavenge free radicals and upregulate antioxidant enzyme expression, as well as inhibit pro-inflammatory molecules [396,397,398]. In terms of AD, the administration of RSV seems to have positive effects in alleviating the symptoms of this neurodegenerative disorder. For instance, some preclinical studies reported its potential effect in improving cognitive impairment, due to its capability to inhibit Aβ aggregation and tau phosphorylation, decrease pro-inflammatory protein expression, and increase Nrf2 nuclear translocation, with the consequent upregulation of HO-1, SOD, CAT, and glutathione peroxidase (GSH-Px) activities [315,317]. It has been shown that its neuroprotective effect could be also due to the RSV-mediated activation of sirtuin 1 (SIRT1), an essential protein for cognitive function and neuronal survival [394]. 

Although these studies underscore the beneficial effects of RSV in managing the disease, due to the few clinical trials and the poor bioavailability of the molecule, as yet, no firm conclusions have been drawn regarding its neuroprotective role and its mechanism of action in humans. For instance, a human study, performed using high doses of RSV, reported a greater reduction in Aβ_1–40_ and Aβ_1–42_ CSF levels in the treated group compared to the baseline [399]; conversely, Gu et al. (employing a lower dose of *trans*-RSV) and Turner et al. found no changes in Aβ_1–42_ CSF and plasma levels in patients treated with this compound [316,400]. These results suggest the importance of further studies with larger samples, different routes of administration, and pharmaceutical formulations, such as nanoencapsulation [401], in order to improve RSV bioavailability and, hence, its effect on cognitive impairment. 

Interestingly, within the diagnostic field, RSV could be used as a potential tool for positron emission tomography (PET), since it is able to map Aβ plaques in the human brain [402].

**Curcumin** (CUR), a yellow phenolic pigment belonging to the curcuminoid family, is widely used as a spice, dye, and food additive, and also as a herbal medicine [403]. Several studies report its powerful biological effects, including its antioxidant, anti-inflammatory, and neuroprotective properties, through which CUR may help to manage different inflammatory and neurological disorders [404,405,406,407,408]. As an antioxidant agent, CUR is able to scavenge free radicals, inhibit lipid peroxidation, and favor HO-1, SOD, and CAT expression; its anti-inflammatory activity is ascribed to a reduction in the levels of several inflammatory cytokines (TNFα, IL-1β, IL-6, IL-8), as well as a reduction in the expression of NF-kB, COX-2, and iNOS [409,410]. 

In the context of AD, numerous in vitro and preclinical studies have shown anti-Aβ-deposition and aggregation, antioxidant, and anti-inflammatory CUR effects. For instance, CUR may inhibit Aβ formation by lowering BACE1 activation and Aβ aggregation (perhaps by directly binding it to the Aβ oligomers), and by promoting Aβ disaggregation [411,412,413]. Furthermore, it has been reported its ability to prevent tau phosphorylation [414] and neuroinflammation by suppressing microglial activation, as well as by reducing TNFα, IL-1β, and IL-6 production [415,416]. In addition, several animal studies report that CUR may improve cognitive function in a dose-dependent manner [417]. 

However, due to CUR’s poor absorption and low bioavailability, AD human studies did not report any improvement in cognitive function or a reduction in Aβ and tau levels [320,418]. In this regard, CUR analogs that were either delivered by nanoparticles or administered with a specific adjuvant could represent possible strategies to improve the effectiveness of this molecule against AD symptoms [319,419,420,421]. Therefore, clinical studies, especially those performed with the support of nanotechnology, are necessary to better understand the properties of CUR and its potential use in AD prevention or treatment. Interestingly, CUR could be used as a tool for AD diagnosis, since it is a fluorochrome that is able to bind Aβ aggregates [422].

**Lignans**, a group of polyphenolic compounds that are abundant in the plant kingdom, possess several biological properties, including anti-inflammatory [423], antioxidant [424], and antitumor activities [425]. Their described neuroprotective role [426] makes them, potentially, natural candidates for AD prevention or treatment. In this regard, some preclinical studies suggested the lignans’ ability to improve learning and memory decline in mice with dementia [427,428]. 

It should be emphasized that among all lignans, especially those isolated from *Schisandra chinensis*, due to their numerous pharmacological effects, they are the most studied compounds, in light of their being a possible therapeutic choice to prevent or slow down the symptoms of AD [322,323,429,430,431,432]. Indeed, the studies that were carried out highlight their ability to enhance cognitive performance by decreasing AChE activity, to reduce Aβ levels by inhibiting BACE1 activity, to prevent tau hyperphosphorylation, as well as to restore SOD and GSH-Px activities. For instance, the possible mechanism by which schisantherin B, one of these lignans, exerts its neuroprotective role could be the activation of the glutamate transporter type 1 (GLT-1) and the consequent inhibition of GSK-3β, involved in tau phosphorylation [429]. A neuroprotective effect of sesamol, sesamin, and other lignans from *Prunus tomentosa* seeds has also been reported [321,433,434]. For instance, sesamol, extracted from sesame oil, was seen to decrease Aβ aggregation, inhibit microglia activation, and reduce TNFα and IL-1β expression in AD mice, with a consequent improvement in memory and learning abilities [433]. 

Overall, considering these encouraging findings in animals, clinical trials are mandatory to investigate the possible effects of lignans in attenuating AD signs and their potential use in AD management. 

### 2.5. Organosulfur Compounds

**Glutathione**. The antioxidant role of the natural tripeptide, γ-l-glutamyl-l-cysteinylglycine, better known as glutathione, is well established [435]. Under normal conditions, redox homeostasis is maintained by the continuous conversion between the oxidized (GS-SG) and the reduced (GSH) forms of glutathione by the enzymes, GPx (which contributes one molecule of glutathione disulfide via oxidation, starting from two molecules of reduced monomeric glutathione) and glutathione reductase (which exploits nicotinamide adenine dinucleotide phosphate (NAPDH) to reduce one GS-SG molecule into two of GSH) [436]. Due to its reducing potential, GSH acts as the main intracellular antioxidant buffer and its synthesis, degradation, transport, metabolism, and interconversion are finely regulated [435,436]. In accordance with a reduction in the total antioxidant capacity during senescence [300], GSH depletion has been observed during aging and age-related diseases, including cardiovascular disease, immune dysfunction, cancer, and neurodegeneration [437,438]. In terms of AD, GSH and GSH-related compounds have been proposed, both as biomarkers and also as therapeutic molecules. It has been reported that both MCI and AD patients show an overall reduction in blood GSH levels, with the intracellular GSH content being particularly decreased in MCI individuals [439]. In line with these data, a comparison between 49 MCI and 19 healthy control subjects revealed that reduced plasma GSH content is associated with worsened cognitive function over a 2-year follow-up period, confirming its potential as a prodromal AD biomarker [440]. Besides peripheral sampling, the possibility of non-invasively monitoring the brain’s GSH content via magnetic resonance spectroscopy imaging is also of interest [441], although the data remain discordant. Accordingly, while significant reductions of cortical GSH levels were reported in female and male AD patients, compared to their healthy counterparts [441], an increase in its content in the anterior and posterior cingulate cerebral areas distinguished MCI individuals from healthy controls in an independent study [442]. Although subsequent studies confirmed a positive correlation between hippocampal/cortical GSH depletion and cognitive impairment [443,444], the high variability, due to inherent sample heterogeneity, differences in the experimental methods, and non-standardized measurement techniques remain a matter of concern [439]. According to Mandal et al., the imaging measurement of cortical GSH is capable of differentially diagnosing MCI and AD with 100% specificity and 91.7% sensitivity, while its hippocampal content diagnoses MCI early, with 87.5% sensitivity and 100% specificity, although the relatively small sample size requires confirmatory studies [443]. Overall, these results underline the importance of further trials involving the time-point monitoring of GSH levels in larger AD, MCI, and control cohorts.

Given the important role of GSH in regulating the total antioxidant capacity, several in vivo studies have investigated the potential of GSH supplementation as a preventative or additional treatment for AD. For example, a three-week oral intake of GSH in the App^NL-G-F/NL-G-F^ knock-in mouse model, which reproduces the features of AD, is sufficient to replenish the brain GSH levels and reduce the hippocampal expression of the OS marker, 4-hydroxynonenal [445]. Similar results were also obtained in preclinical AD models following treatment with different GSH-related molecules, including the GSH mimetic D609, the GSH precursor, S-adenosyl methionine (SAM), and a GSH analog that is resistant to γ-glutamyl-transpeptidase activity, which usually degrades GSH [446,447,448]. The reported improvements include diminished OS-related products, malondialdehyde and protein carbonyls, the potentiation of the antioxidant machinery by increasing GSH levels and SOD activity, the prevention of insoluble Aβ deposition, and improved spatial learning and memory processes [446,448]. Notably, the re-establishment of OS homeostasis was often accompanied by reduced microgliosis and neuroinflammation, mediated by an increased expression of the anti-inflammatory cytokine, IL-10, at the expense of the pro-inflammatory cytokines (i.e., TNFα, IL-6, and IL-1β), as shown by the in vitro and in vivo data [445,449]. As a further confirmation of the importance of GSH metabolism in modulating neurodegeneration, the restoration of GPx in the cortical neurons and Aβ_1–42_-treated mice blocked neurotoxicity, attenuated memory decline, and activated the PKC βII-mediated ERK pathway, which is implicated in AD onset [450,451]. Besides neurons, the protective effect exerted by GSH on the brain endothelial cells is also of interest for neurodegenerative diseases, since it counteracts H_2_O_2_-mediated NO, ROS, and 8-hydroxy-2′-deoxyguanosine production, strengthens the tight junction proteins, and promotes the activity of the antioxidant Nrf2 pathway [452].

Despite yielding promising data, the limited uptake of GSH or GSH-precursor supplementations, combined with the inherent age-related GSH homeostasis dysregulation, calls for novel prodrug formulations, innovative GSH-related compounds, and delivery optimization [449].

**Allicin, allyl sulfide indoles, and allylcysteines**. Onion and garlic are excellent sources of a variety of sulfur-containing compounds with known antioxidant, immunomodulatory, antitumoral, and anti-inflammatory properties, including allicin, alliin, allyl sulfide indoles, and allylcysteines [453,454,455,456]. Mainly found in fresh and aged garlic extracts, these compounds have been shown to prevent neurotoxicity by reducing cerebral Aβ deposition while disaggregating preformed fibrils in a dose-dependent manner [457,458,459,460,461]. Improved hippocampal memory has also been reported in transgenic AD mice supplemented with aged garlic extract [462], which displays a higher total antioxidant capacity compared to fresh garlic extract [463]. Accordingly, the pretreatment of cognitively normal adult male rats with aged garlic extract showed improved short-term recognition and working and reference memory, compared to their untreated counterparts [464,465]. These benefits are mediated by reduced microgliosis, a diminished expression of the pro-inflammatory cytokine, IL-1β, attenuated cholinergic neuronal loss, and increased hippocampal levels of the vesicular glutamate transporter 1 and glutamate decarboxylase [454,464,465], which regulate glutamate transport and metabolism and are normally downregulated in AD [466,467]. Similar results were obtained upon the administration of S-allyl cysteine (SAC), one of the main ingredients of aged garlic extract [455,468]. Elsewhere, studies showed that the SAC pretreatment of streptozotocin-infused mice (an animal model of diabetes) drastically improved cognitive function, antioxidant capacity (measured by GSH, GPx, and glutathione reductase levels) and prevented apoptosis (assessed by DNA damage and Bcl-2 and p53 expression) [469]. However, the aged garlic extract’s capacity to protect against synaptic degeneration, inhibit Aβ and neurofibrillary tangle deposition, reduce neuroinflammation, and counteract ROS formation appears to be greater than SAC alone, likely suggesting a synergistic effect among the various sulfur-containing compounds found in aged garlic extract [470].

Another main constituent of garlic known for its phytochemical properties is allicin, a hydrophobic molecule produced from alliin when garlic is chopped or damaged [471]. Being a reactive sulfur species (RSS), its antioxidant activities include Nrf2 upregulation, ROS quenching, and neuroinflammation dampening [471,472]. Moreover, it exerts an inhibitory effect against various pro-inflammatory and pro-oxidative signaling behaviors, including the TLR4/myeloid differentiation primary response 88 (MyD88)/NF-kB, and the Jun N-terminal kinase (JNK), p38 MAPK, and AChE pathways [471,472]. To date, in vivo studies prove that allicin supplementation improves cognitive function while reducing Aβ expression and accumulation in various preclinical AD models, including aluminum chloride-treated rats, APP/PS1 mice, and Aβ_1–42_ treated mice [473,474,475,476]. Mechanistically, these benefits are mediated by oxidative homeostasis re-establishment through increased antioxidant potential versus OS, reduced pro-inflammatory cytokine (TNFα, IL-6, and IL-1β) release, improved mitochondrial function, neurotransmitter (GABA, dopamine, serotonin, glutamate, and norepinephrine) concentration, and decreased tau-phosphorylation [473,474,475,476]. Interestingly, allicin is also protective against endoplasmic reticulum (ER) stress-mediated neurotoxicity, as it increases, for instance, the hippocampal expression of the ER-resident kinase involved in the ER stress response, PERK (protein kinase RNA-like endoplasmic reticulum kinase), together with its substrate, Nrf2 [476]. Still, the lack of data in humans and the need for a larger sample size and validation studies constrain clinical development.

**α-lipoic acid.** α-lipoic acid (LA), or thioic acid, is a natural antioxidant that is derived from caprylic acid and belongs to the group of organosulfur compounds [477]. Thanks to its anti-inflammatory, antioxidant, neuroprotective, and anti-amyloidogenic properties, LA has recently been considered as a treatment agent for various neurological conditions, including neurodegeneration [477,478]. In terms of AD, preclinical evidence shows that LA administration to mouse and rat models of the disease increases memory and learning ability, reduces cognitive decline, and ameliorates motor functions, compared to their untreated counterparts [479,480,481,482,483,484]. These benefits are mediated by decreased oxidative stress (measured by lower levels of MDA in favor of the antioxidant enzymes’ enhanced expression of GSH, SOD-1, and CAT), reduced neuronal death, diminished neuroinflammation (measured by the number of reactive astrocytes), and impaired Tau hyperphosphorylation [479,480,481,482]. Notably, even better results are obtained upon pairing the intake of LA with physical exercise or with the administration of anti-inflammatory drugs, such as ibuprofen [482,483,484,485]. Despite these improvements, however, some evidence reports that the administration of LA to AD mice has no impact on amyloid pathology and may even reduce the lifespan, suggesting that more studies are needed to better evaluate LA’s safety and efficacy [481,482,483]. When tested in humans, results from an open-label study that was conducted on 43 patients showed that daily LA intake significantly lowered disease progression in both MCI and AD subjects, thus confirming its neuroprotective properties [486]. Moreover, data obtained from a randomized controlled trial involving 39 AD patients confirmed slightly slower AD progression in the group administered with LA, with better outcomes reported upon combining LA with omega-3 fatty acids intake [487]. Overall, these data point to LA as a promising anti-AD agent, but further studies are needed to further assess its therapeutic efficiency.

### 2.6. Mitochondria-Targeted Antioxidants

Impaired glucose metabolism, oxidative stress, and mitochondrial dysfunction are all conditions that characterize human aging and age-related disorders [488]. Concerning neurodegeneration, mitochondria dysfunction contributes to AD pathophysiology through insufficient ATP biogenesis, diminished oxidative phosphorylation, imbalanced mitochondria biogenesis-mitophagy, and impaired antioxidant defenses [489,490,491,492]. To date, several mitochondria-targeted antioxidants have been proposed as alternative therapeutic molecules in AD, including CoQ_10_, SS31, mitoquinone (MitoQ), methylene blue (MB), and SkQs (Table 2) [493,494,495,496,497,498,499,500,501,502,503,504,505,506,507,508,509,510,511,512,513,514].

Among mitochondria-targeted molecules, **CoQ_10_** is a lipid-soluble antioxidant belonging to the electron transport chain, which plays a key role in mitochondrial oxidative phosphorylation, inflammation, metabolism, and gene expression [515]. When it is lacking, CoQ_10_ has been linked to several disorders, such as cancer, fibromyalgia, cardiovascular disease, diabetes, and neurodegeneration [516]. In the context of AD, CFS samples obtained from AD patients display an enrichment in oxidized CoQ_10,_ compared to those from aged-matched healthy controls, thus suggesting that mitochondrial oxidative damage may be a helpful parameter for disease diagnosis [517]. However, the use of CoQ_10_ as an early or prodromal AD biomarker remains unlikely, as no association between serum CoQ_10_ levels and MCI was reported in the randomized controlled trial of the ActiFE study [518]. Nevertheless, despite the diagnostic potential of CoQ_10_ remaining unclear, several preclinical studies are consistent in showing a therapeutic advantage from CoQ_10_ intake (Table 2). Accordingly, various rat and mouse models of AD CoQ_10_ administration resulted in improved memory, behavior, and cognitive performance, as well as the prevention of AD-associated hippocampal long-term potentiation impairment [503,506,509,512]. These effects are mediated by increased antioxidant capacity (GSH, CAT, and SOD), improved mitochondrial respiratory enzyme activity, and reduced levels of lipid peroxides, serum MDA, and brain protein carbonyls, which are considered the hallmarks of OS [503,505,506,509,511,512]. Notably, amyloid pathology (as measured by the brain levels of Aβ_1–42_ and the amyloid plaque burden) was consistently decreased across studies, indicating a promising role of CoQ_10_ in preventing Aβ-induced neurotoxicity [503,505,506,514]. Although the free radical scavenging activity of CoQ_10_ may appear as the only mediator of these benefits, the anti-apoptotic and anti-inflammatory properties of this antioxidant are equally important in the AD context [503,519]. In this respect, there is evidence that CoQ_10_ promotes neurogenesis through the PI3K pathway and limits neuroinflammation by decreasing the levels of the pro-inflammatory molecules, COX-2, prostaglandin E_2_ (PGE2), NF-kB, TNFα, IL-6, IL-1β, and apolipoprotein E [512,520,521,522]. In line with this evidence, the treatment of AD rats with a combination of CoQ_10_ and minocycline, a microglial inhibitor, further ameliorated AD symptomatology and improved cognitive performance [512]. Other combinations, such as CoQ_10_ with vinpocetine (a synthetic derivative of the *Vinca* alkaloid, vincamine) and physical/mental practice, or CoQ_10_ with omega-3 intake are also promising [504,523]. In the latter case, significant improvements in the OS, inflammatory, amyloidogenic, and cholinergic parameters were reported in hypercholesterolemia-induced AD rats and were correlated with increased memory and brain functions [504].

Despite the encouraging therapeutic potential of CoQ_10_, poor brain accumulation remains a matter of concern. Indeed, although CoQ_10_ can pass through the BBB, its opposite transport to the blood may limit its therapeutic efficacy [524]. In this respect, CoQ_10_ analogs that passively cross the BBB, such as idebenone, have been reported to reduce tau hyperphosphorylation, caspase 3 activity, and the amyloid burden through the upregulation of the α-secretase and neprilysin enzymes, which reduce the Aβ production/burden [508]. Moreover, a new water-dispersible formulation, based on ubisol-Q_10_, in which an amphiphilic molecule is linked to CoQ_10_, enabling the formation of nanomicelles, has recently been proven to be effective in reducing Aβ plaques and increasing long-term and spatial working memory in transgenic AD mice [507]. Compared to CoQ_10_, the ubisol-Q_10_-mediated upregulation of the autophagy pathway may explain its potent neuroprotection, even if delivered after, as well as before, or concomitantly with, neurotoxin exposure [510,525]. Currently, new delivery methods are being studied to increase its targeting and efficiency. For example, Sheykhhasan et al. recently showed that CoQ_10_ delivery via adipose-derived stem cell exosomes is more efficient than CoQ_10_ alone in stimulating the memory and increasing the hippocampal expression of BDNF and SRY-box transcription factor 2 (SOX2) in AD rats, while additional studies might be published shortly [513].

Overall, although the use of CoQ_10_ for diagnostic purposes remains unclear, its therapeutic potential has been repeatedly proven in different preclinical models, paving the way for clinical trials.

**MitoQ** is a lipid-soluble mitochondria-targeted molecule composed of the CoQ_10_ ubiquinone, which is linked to the lipophilic triphenyl phosphonium (TPP) cation, making it a powerful antioxidant [526,527], although its effect is only directed toward the mitochondrial membrane [497]. When tested in several in vitro models of AD, MitoQ’s ability to limit ROS generation, re-establish the optimal mitochondrial membrane potential, and promote mitochondrial function (as measured by reduced cyclophilin D and increased peroxiredoxins) has been proven capable of stimulating neurite outgrowth, preventing tau-dimerization, and protecting against Aβ-induced neurotoxicity [496,528,529]. In line with these data, nematode models of AD treated with MitoQ showed an extended lifespan and increased mitochondria cardiolipin [497], an essential component of the mitochondrial membrane [530]. Similarly, 3xTg AD mice that received MitoQ in drinking water for 5 months exhibited a reduction in astrogliosis, synapse loss, Aβ aggregation, microglial activation, and tau hyperphosphorylation, which was associated with improved memory ability and delayed death [495,496,531]. However, despite promising preclinical data, to date, no clinical studies have been reported on MitoQ efficacy in AD patients, thus emphasizing the need for further investigations.

**SkQ1** is a mitochondria-targeted antioxidant belonging to the class of SkQs molecules, which are composed of a lipophilic cation, a plastoquinone molecule that functions as an antioxidant moiety, and an alkane linker region [300,502]. To date, data obtained mainly from preclinical studies have demonstrated the effectiveness of SkQ1 against aging and AD [532]. Indeed, it has been observed that hippocampal slices obtained from rats pre-treated with SkQ1 show the restored induction of LTP, an indicator of synaptic plasticity, compared to slices treated with Aβ alone [502]. In vivo, lessened neurodegeneration and reduced anxiety, together with improved locomotor and exploratory activity have been reported in OXYS rats upon receiving an SkQ1 intake, although learning ability and neurogenesis were not promoted [498,501,533]. These beneficial effects are mediated by a reduction in mitochondrial and synaptic damage, hippocampal Aβ_1–40_ and Aβ_1–42_ accumulation, neuronal loss, and tau hyperphosphorylation, together with enhanced levels of the synaptic proteins (which regulate the release of neurotransmitters) and neurotrophic factors [499,533]. In addition, the anti-inflammatory properties of SkQ1 help to slow down AD progression by inhibiting the p38 MAPK signaling pathway and shifting the activated microglia toward a resting state, thus limiting the neurotoxicity [498,500]. Outside the brain, there is evidence that SkQ1 administration prevents the development of retinopathy due to Aβ accumulation, but more data are needed to better assess its beneficial role against retinal damage [501,534].

### 2.7. Minerals

Since the pathogenesis of AD appears to be closely related to the impact of OS on the promotion of neurodegenerative mechanisms, the role of minerals in AD has been explored in the literature since they are essential for the antioxidant activity of many enzymes [535,536]. In fact, the detection of a reduced plasmatic concentration of trace elements, such as selenium, zinc, iron, and copper, in AD patients may suggest a possible target of the pathology that could, therefore, be partially counteracted by means of a specific dietary intervention in these individuals [537,538]. In this regard, several studies reported the numerous beneficial effects of minerals against AD (Table 3) [539,540,541,542,543,544,545,546,547,548,549,550,551,552,553,554,555,556,557,558,559,560,561,562,563,564,565,566,567,568,569,570,571,572,573,574,575,576,577,578,579,580,581,582,583].

**Selenium.** The importance of selenium (Se) is mainly related to its presence at the active center of the so-called selenoproteins, the neuroprotective role of which is mainly related to their antioxidant activity via the reduction of ROS and RNS, the regulation of calcium transport, and their anti-inflammatory effects [584]. Se deficiency was observed to be in direct correlation with the reduction of GPx in patients with AD [585]. Evidence from the literature shows lower plasma levels of Se, both in elderly patients with general cognitive decline and in patients diagnosed with MCI or AD, as well as in the brain and the CSF of AD individuals [585,586,587,588]. From these considerations, there follows the hypothesis of Se supplementation as a supportive therapy for AD; in fact, its implementation in patients via sodium selenate, which elevates Se levels in the CSF, or via Se-rich nanoparticles, which reduce ROS and, consequently, counteract Aβ deposition, was observed to have a potential therapeutic role for AD [540,589]. In addition, studies in rats pointed to the existence of molecular mechanisms supporting the use of Se supplementation to counteract AD: (i) Se prevents the reduction of antioxidant enzymes such as GPx, (ii) it reduces membrane permeability for Ca^2+^ by acting as an NMDA receptor antagonist, (iii) it contributes to rising membrane phospholipid levels, which act as an indirect marker of the synaptogenetic process [541,542,543]. All these effects were also noticed in mice treated with the bioactive organic form of selenomethionine (Se-Met), leading to better cognitive performance; additionally, Se-Met was observed to decrease the tau levels, both in its total amount and in its phosphorylated form, to regulate the autophagic process, and to be able to reverse synaptic dysfunction [544,545,546,547]. Furthermore, sodium selenate may represent a promising supportive therapy because of its ability to decrease tau aggregation and, more generally, to modify the cortical proteome contrasting AD in murine models [548,549,590]. Finally, Se also seems to possess beneficial effects in co-supplementation with resveratrol, folic acid, and probiotics, with which the antioxidant effects appear to be even more significant in comparison to Se when administered alone [550,551,552,591].

The effects of Se, particularly when referring to selenoprotein P, also seem to have an impact on zinc balance, the alteration of which was observed to be linked to tau hyperphosphorylation in SELENOP1 knockout mice [592].

**Zinc.** There is some evidence in the literature supporting the finding that zinc (Zn) can be considered a neuroprotective factor against AD, which is indeed often accompanied by reduced levels of Zn itself. In particular, this trace element was observed to be avidly bound by amyloid plaques, exerting an antioxidant effect, and its supplementation was linked to a reduction in monomeric Aβ and to significant inhibiting activity on anticholinesterase enzymes in the in vitro studies [554,555,556]. The in vivo studies in murine models show that Zn treatment, delivered by nanoparticles, is associated with a downsizing of the plaques [557]. Furthermore, Zn was demonstrated to effectively counteract the neural damage induced by other metals, such as aluminum (Al) and copper (Cu). In more detail, zinc sulfate administration is able to restore normal levels of tau, APP, and α-synuclein and to ameliorate the histological architecture altered by Al in mice; furthermore, Zn counteracts the toxic effects of Cu (which fuels ROS formation) at the brain level, limiting its intestinal absorption in AD patients [558,559,560,593]. Zn was also observed to alleviate cognitive impairment in rats when co-administered with Se, as these substances seem to stabilize the mitochondrial membranes and protect them from oxidative stress by increasing the SOD and GPx levels; in addition, Zn, together with Se and fish oil (eicosapentaenoic acid and docosahexaenoic acid), inhibits APP cleavage [561,562].

Interestingly, in vivo and in vitro studies suggest that an excess of Zn can represent a risk factor for the development of AD, as it promotes APP expression, β-secretase cleavage, and Aβ deposition and aggravates tauopathy, resulting in learning and memory impairment [594,595]. Based on this rationale, Zn chelation could be considered a therapeutic strategy in this condition: in fact, clioquinol has been reported to reduce Aβ plaques [563,596]. The same mechanism seems to be exploited by the S100A6 protein, which is synthesized in astrocytes surrounding Aβ deposits and shows a disaggregating capacity due to Zn sequestration [564].

Notwithstanding, the literature still shows conflicting data; for instance, chronic Zn supplementation did not modify Aβ and tau deposition in a study on murine models [597]. Therefore, further studies are needed to solve this controversy regarding the role of Zn in AD pathophysiology and its homeostasis.

**Iron.** Iron excess and its local deposition, particularly in the basal ganglia, was observed to have a pro-inflammatory and pro-aggregating effect, leading to Aβ plaque formation; additionally, its accumulation impacts cerebral perfusion and, also, on synapse plasticity via a decrease in the levels of furin, which is an enzyme that regulates the maturation of the BDNF and other proteins linked to synaptogenesis [598,599,600,601]. These observations support the hypothesis that iron chelators could represent a valid therapeutic tool, as was also suggested by experiments demonstrating that deferoxamine and deferiprone (iron chelators) are able to reduce Aβ aggregation in the brains of murine AD models and attenuate cognitive impairment [565,566,567,568]. The same mechanism is exploited not only by well-known antioxidants, such as hesperidin and naringin, but also by coumarin, curcumin, capsaicin, and S-allylcysteine, which decrease the Aβ burden by binding iron [569,570,571]. New formulations of iron chelators that target multiple factors involved in AD pathogenesis include M30, a compound that also decreases APP expression and tau phosphorylation, and aroyl nicotinoyl hydrazones (especially SNH6), which also reduce oxidative stress via the elevation of NAD^+^ levels and the subsequent implementation of sirtuin activity, which results in protection against axonal damage [572,573,574]. Another strategy to contrast with local iron deposition consists of using chelators, such as PBT434 (which hinders iron uptake by the endothelial cells within the BBB by binding the metal in the interstitium and, at the same time, stimulating iron efflux via an increase in ferrous iron in the intracellular compartment), and by using nanoparticles as carriers of the chelators themselves, which can then cross the BBB [575,576].

Despite all these findings, it is interesting to note that the literature also reported the beneficial effects related to iron administration, which seems to inhibit Aβ_1–42_ accumulation in mice models, thus suggesting the need for a balanced amount of this trace element, in order to promote a healthy condition [577].

**Copper.** Another contributor to oxidative stress is Cu, the overload of which was observed to determine tau hyperphosphorylation and, consequently, the formation of neurofibrillary tangles in AD patients. In addition, Cu tends to bind certain regions of Aβ when released following APP processing, exacerbating fibril deposition [602,603]. The pathogenic role of Cu is also supported by the finding that the supplementation of this trace element deteriorates the mitochondrial functioning in the hippocampus and induces axonal damage in AD mice models by means of an altered phosphorylation of the CAMK2α and ERK1/2 kinases [604]. Based on this rationale, molecules with Cu chelation activity, such as PA1637 and TDMQ20, have been developed and tested in murine models, showing the capacity to ameliorate memory deficits and generally improve cognitive and behavioral performance, respectively [578,579]. Interestingly, other well-known antioxidants, such as the flavonoid, fisetin, show the ability to neutralize the negative effects of Cu overload by binding this metal with iron [580]. Despite the cognitive improvements, the literature reports the limited impact of Cu chelators on the molecular mechanisms underlying tau neuropathology [581]. However, the consideration that AD is a multifactorial disease leads current researchers to focus on the development of multi-target and multi-functional ligands. Within this context, compounds characterized by the ability to combine the beneficial effects of Cu chelators with other activities, such as acetylcholinesterase and butyrylcholinesterase inhibition, could represent a promising therapeutic strategy [582,583].

## 3. Discussion

With the current exponential increase in the elderly population, the search for predictive and prognostic biomarkers for AD’s onset and progression, as well as the implementation of innovative approaches to prevent and treat neurodegeneration, are of the utmost importance. Notably, lifestyle and nutrition have turned out to be crucial regulators of the human lifespan [605,606,607,608]. It was shown that physical and mental exercise, dietary habits, antioxidant intake, vitamin supplementation, and protection from pesticide exposure reduce the risk of developing sporadic AD later in life [609,610,611]. Although not effective enough as therapeutics, these tools should be considered lifelong preventative approaches to antagonize aging and age-related neurodegeneration, with profound social implications [612]. Nevertheless, most single-factor interventions that have been tested so far turned out to be ineffective, likely due to the complex and multifactorial etiology of AD [612]. In this context, a multivariate preventative intervention, targeting several disease-causing mechanisms simultaneously, would probably be more beneficial [612,613]. Accordingly, the results from the randomized controlled FINGER trial showed that multidomain lifestyle interventions could improve cognition among old people who are at high risk of developing dementia, encouraging further investigation [612,613].

OS has long been considered to participate actively in the pathophysiology of AD, making it an excellent diagnostic and therapeutic tool [614]. For this reason, in this narrative review, we have carefully summarized the potential of carotenoids, vitamins, flavonoids, non-flavonoids, organosulfur compounds, mitochondria-targeted antioxidants, and minerals to serve as biomarkers, as well as antioxidant buffers against AD onset and progression. However, despite the promising results, some limitations still exist and need to be addressed before moving forward to the clinical application. For instance, one of the greatest concerns is the definition of the duration of the therapeutic treatment. Indeed, while most studies limited the antioxidant administration to a defined time window, evidence of the effect of long-life treatment remains poor. In this context, since OS actively participates in the AD etiology [615], an early and lasting intervention could be more effective, if not even preventive, than a late and heavy treatment [616]. Moreover, the metabolic changes that normally occur during aging [617] should be taken into account when designing the dosage and administration window. Nevertheless, it is often difficult to clearly identify the optimal dosage of antioxidants, which depends not only upon the defined treatment but also on dietary and supplement intake. In this respect, it has been reported that antioxidants can act as pro-oxidants when accumulated in excessive amounts [300,618], underlying the importance of patient-to-patient evaluation. This holds particularly true when considering the fact that older people make use of several chronic medications, which may end up interfering with the expected activity of the recommended supplementation. If antioxidant combinations are then considered, careful assessment of synergism and antagonism among various compounds should be conducted, as the simultaneous administration of several antioxidants does not always represent the best option [619]. Even when taking into account each compound alone, storage conditions and environmental factors may greatly affect the reducing potential [620], partially explaining the inconsistency in the data that are sometimes reported by independent studies.

## 4. Conclusions

Considering clinical translation, a major limitation of this work remains the lack of human studies for most of the antioxidant compounds presented in this review. The abundance of data coming from murine or in vitro approaches compared to human investigations hinders clinical development. Further research should focus on identifying the optimal dosage and treatment window, so as to plan large randomized clinical trials aimed at better assessing the diagnostic and therapeutic potential of these innovative strategies.

## Figures and Tables

**Table 1:** Effects of non-flavonoid treatment in several experimental studies.

Non-Flavonoid	Experimental Model	Treatment Duration	Treatment Effects	Reference
**Ferulic acid**	In vivo—PSAPP transgenic mouse model	6 months	Amelioration of behavioral performance Reduction in Aβ deposits, as well as in Aβ_1–42_ and Aβ_1–40_ abundance by the inhibition of BACE1 activity Attenuation of neuroinflammation (↓GFAP and Iba1 levels, as well as ↓TNFα and IL-1β mRNA expression) and OS (↑SOD1, CAT and GPx1 mRNA expression)	[302]
	In vitro—Aβ_1–40_ -damaged PC12 cells	30 min	Prevention of cell death Reduction in intracellular ROS Inhibition of Aβ_1–40_ aggregation	[303]
	In vivo—Aβ_1–40_ -induced mouse model	21 days	Improvement in cognitive abilities by increasing SOD and ChAT activity and by decreasing AChE activity Attenuation of lipid peroxidation (↓MDA levels)	
**Caffeic acid**	In vitro—Aβ_25–35_ -damaged PC12 cells	1 h	Protection against Aβ-induced toxicity by inhibiting OS, calcium influx, and tau hyperphosphorylation	[304]
	In vivo—Aβ_25–35_ -induced mouse model	2 weeks	Improvement in spatial cognitive and memory functions Inhibition of lipid peroxidation (↓MDA levels) and NO formation	[305]
	In vivo—Aβ_1–42_ -induced mouse model	10 days	Decreasing in neuronal apoptosis (↓caspase 9) and neuroinflammation (↓GFAP and Iba1 expression) Improvement in learning and memory Attenuation of OS by inducing the Nrf2/HO-1 signaling pathway	[306]
**p-Coumaric acid**	In vitro—Aβ_25–35_ -damaged PC12 cells	1 h	Attenuation of Aβ_25–35_ -induced toxicity, through reduction of neuroinflammation (↓iNOS and COX-2), via downregulation of NF-kB and MAPKs pathways	[307]
	In vivo—D-galactose mouse model	42 days	Amelioration of cognitive performance by decreasing AChE levels Attenuation of OS (↑SOD and GSH) and neuronal apoptosis (↓Caspase3) Reduction in NF-kB and BACE1 levels	[308]
	In vitro—SH-SY5Y cells	4 h	Attenuation of apoptotic cell death (↓Caspase3) Reduction in ROS accumulation, as well as in cytochrome c release into the cytosol	[309]
	In vivo—Aβ_1–42_ -induced rat model	2 weeks	Amelioration of learning and memory deficits and neuronal apoptosis	
**Gallic acid**	In vivo—AlCl_3_ -induced rat model	60 days	Amelioration of spatial memory and learning deficits Reduction in neurofibrillary tangles and amyloid plaques Increase in CAT, GSH, and SOD activity, and a decrease in MDA and NO contents	[310]
	In vivo—APP/PS1 transgenic mouse model	30 days	Amelioration of spatial memory and learning impairments Inhibition of Aβ aggregation Reduction in neuroinflammation (↓GFAP) and increase in synaptic strength	[311]
**Ellagic acid**	In vivo—APP/PS1 transgenic mouse model	60 days	Improvement in learning and memory abilities Decrease in Aβ production by reduction of BACE1 levels Inhibition of tau phosphorylation by modulation of Akt/GSK-3β signaling pathway	[312]
	In vivo—Aβ_25–35_ -induced rat model	1 week	Amelioration of learning and memory deficits Attenuation of OS, by increasing antioxidant defense (CAT, GSH), and neuroinflammation (↓NF-kB) Reduction in AChE activity	[313]
	In vivo—AlCl_3_ -induced rat model	4 weeks	Increase in SOD and GSH levels Attenuation of lipid peroxidation Reduction in neurofibrillary tangles and neuritic plaques	[314]
**Resveratrol**	In vivo—SAMP8 mouse model	7 months	Increase in lifespan Activation of AMPK signaling pathways and increase in SIRT1 levels Reduction in cognitive impairments as well as in Aβ burden and tau phosphorylation	[315]
	In vivo—SAMP8 mouse model	15 days	Improvement in learning abilities Increase in the activity of SOD, GSH-Px, and CAT through the Nrf2/HO-1 signaling pathway Decrease in MDA content	[316]
	In vivo—30 AD patients	52 weeks	Not statistically significant changes in Aβ_1–40_ levels in blood and CSF Reduction in brain volume at 52 weeks Decrease by 46% in MMP-9 levels in CSF	[317]
**Curcumin**	In vivo—Transgenic mouse model APPSw	6 months	Suppression of inflammation (↓GFAP, and IL-1β) and oxidative damage Decrease in insoluble and soluble amyloid as well as in plaque burden	[318]
	In vivo—Aβ-induced rat model	4 days	Improvement in learning and memory performance Reduction in OS parameters (ROS formation, lipid peroxidation, and ADP/ATP ratio) as well as in amyloid plaques	[319]
	In vivo—48 AD patients	24 weeks	Not effective on CSF and plasma AD markers, including Aβ_1–42_, tau, and p-tau	[320]
**Lignans**	In vivo—Scopolamine-induced rat model	2 weeks	Improvement in rat behaviors Alleviation of OS (↑CAT and SOD) and lipid peroxidation (↓MDA) Decrease in AChE levels	[321]
	In vivo—Aβ_1–42_ -induced mouse model	5 days	Improvement in learning and memory abilities by reduction of ChE total levels and increase of SOD, GSH-Px activity as well as GSH content Attenuation of memory impairment	[322]
	In vivo—Aβ_1–42_ -induced mouse model	4 days	Reduction in Aβ_1–42_ levels by inhibition of β-secretase activity Inhibition of AChE activity and reduction of GSH levels	[323]

Abbreviations: Aβ: amyloid beta peptide; AChE: acetylcholinesterase; AD: Alzheimer’s disease; ADP/ATP: adenosine diphosphate/adenosine triphosphate; Akt/GSK-3β: protein kinase B/glycogen synthase kinase-*3β;* AlCl_3_: aluminum chloride; AMPK: AMP-activated protein kinase; BACE1: beta-site APP cleaving enzyme 1; CAT: catalase; ChAT: choline acetyltransferase; ChE: cholinesterase; COX-2: cyclo-oxygenase-2; CSF: cerebrospinal fluid; GFAP: glial fibrillary acidic protein; GPx1: glutathione Peroxidase 1; GSH: glutathione; GSH-Px: plasma glutathione peroxidase; Iba1: ionized calcium-binding adapter molecule 1; IL-1β: *interleukin 1 beta;* iNOS: inducible nitric oxide synthase; MAPK: mitogen-activated protein kinase; MDA: malondialdehyde; MMP-9: matrix metallopeptidase 9; NF-kB: nuclear factor kappa B; NO: nitric oxide; Nrf2/HO-1: nuclear factor erythroid 2-related factor/Heme Oxygenase-1; OS: oxidative stress; ROS: reactive oxygen species; SAMP8: senescence-accelerated mouse prone 8; SIRT1: sirtuin 1; SOD: superoxide dismutase; SOD1: superoxide dismutase 1; TNFα: tumor necrosis factor alpha; ↑: increase; ↓: decrease.

**Table 2:** Preclinical and clinical studies on mitochondria-targeted compounds in AD.

Compound	Experimental Model	Treatment	Results	Ref.
**MitoQ**	3xTg-AD female mice	1: 4 mix of 100 μM MitoQ + β-cyclodextrin for 5 months in drinking water	↑ memory, lifespan ↓ brain OS, astrogliosis, Aβ accumulation, tau hyperphosphorylation, microglial proliferation, caspase activation	[495,496]
	*Caenorhabditis elegans* overexpressing human Aβ	1 µM MitoQ in NGM agar and *Escherichia coli* OP50-1	↑ lifespan, healthspan, electron transport chain function	[497]
**SkQ1**	OXYS male rats (12-month-old)	250 nM SkQ1/kg daily for 6 months	↑ resting/activated microglia ratio, learning, memory, synaptic function, neurotrophic supply, locomotor, and exploratory functions ↓ inflammation, neurodegeneration, neuronal loss, synaptic damage, p38 MAPK signaling, AD progression, tau hyperphosphorylation, Aβ_1–42_	[498,499,500,501]
	Male Wistar rats	One i.p. injection of 250 nM SkQ1/kg	↑ neuroprotection ↓ Aβ-induced OS	[502]
**CoQ_10_**	AlCl_3_ treated rats	Biotin (2 mg/kg), CoQ10 (10 mg/kg) for 60 days	↑ insulin signaling ↓ inflammation	[503]
	Hypercholesterolemic rats	10 mg/kg for 30 days (oral)	↑ memory, cholinergic function ↓ brain OS and inflammation, amyloidosis	[504]
	C65/Bl6 mice	10 g/kg for one month	↓ brain OS measured by protein carbonyls	[505]
	Tg19959 mice	3 months of 0.4% CoQ_10_ in chow or 5 months of 2.4% CoQ_10_ in chow	↑ cognitive function (Morris water maze test) ↓ amyloid pathology, brain OS measured by protein carbonyls	[506]
	Male Wistar rats	50 mg/kg of CoQ_10_ daily for 6 weeks (3 before and 3 after AD induction)	↑ EPSP slope and population spike amplitude ↓ serum malondialdehyde, OS	[509]
	Female mice overexpressing presenilin 1-L235P	1200 mg/kg of CoQ_10_ daily for 60 days	↑ SOD activity ↓ MDA levels, cortex Aβ burden	[511]
	Male Sprague–Dawley rats	20 and 40 mg/kg for 21 days	↑ SOD, CAT, GSH, mitochondrial respiration ↓ transfer latency, AChE activity, TNFα, LPO, nitrite	[512]
	Wistar rats	CoQ_10_-loaded ADSCs-exosomes	↑ Cognition, memory, hippocampal BDNF and SOX2	[513]
	APP/PS1 transgenic mice	1200 mg/kg CoQ_10_ daily for 60 days	↓ Aβ plaque burden	[514]
**Ubisol-Q_10_**	TgAPEswe, PSEN1dE9 mouse	6 mg/kg Ubisol-Q_10_ daily for 18 months	↑ long-term and working spatial memory ↓ circulating Aβ, Aβ plaque formation	[507]
	Male APP/PS-1 mice	200 μg/mL of Ubisol-Q_10_ in drinking water for 18 months	↑ cortical beclin-1 and JNK1, autophagy	[510]
**Idebenone**	5xFAD mice	i.p. injection of 100 mg/kg/day for 14 days	↑ NEP, α-secretase ADAM17, tau hyperphosphorylation, total tau ↓ Aβ plaque number, RAGE/caspase 3 signaling	[508]

Abbreviations: 3xTg-AD mice: triple transgenic Alzheimer’s disease mice; AChE: acetylcholinesterase; ADAM17: ADAM metallopeptidase domain 17; ADSCs: adipose-derived stem cells; APP/PS1: amyloid precursor protein/presenilin 1; BDNF: brain-derived neurotrophic factor; CAT: catalase; CoQ10: coenzyme Q10; EPSP: excitatory postsynaptic potential; GSH: glutathione; i.p.: intraperitoneal; JNK1: Jun N-terminal kinase 1; LPO: lipid peroxidation; MDA: malondialdehyde; MitoQ: mitoquinone mesylate; NEP: neprilysin; NGM: nematode growth medium; OS: oxidative stress; OXYS rats: an experimentally renowned model of inbred strains of rats for a range of degenerative diseases in man; RAGE: receptor for advanced glycation end-products; SkQ1: plastoquinonyl-decyltriphenylphosphonium; SOD: superoxide dismutase; *SOX2*: SRY-Box Transcription Factor 2; TgAPEswe, PSEN1dE9: double transgenic mouse model of Alzheimer’s disease; TNFα: tumor necrosis factor alpha; ↑: increase; ↓: decrease.

**Table 3:** Preclinical and clinical studies on the therapeutic use of minerals or ion chelators for AD.

Study Design	Treatment	Results	Conclusion	Reference
CSF of AD patients	0.32 or 10 mg sodium selenate oral supplementation 3 times daily	↑ Se CSF levels	Sodium selenate as a possible therapeutic tool against AD	[539]
STZ-induced male rats	0.4 mg/kg Se nanoparticles oral administration daily for one month	↓ ROS ↓ Aβ deposition	Selenium nanoparticles to contrast AD pathogenesis	[540]
ICV-STZ rats	0.1 mg/kg intraperitoneal sodium selenite for 7 days	↓ reduction of GPx	Sodium selenite as a possible supportive approach to treat SDAT	[541]
Hippocampal and dorsal root ganglion neuronal cultures from 1 or 1.5 mg/kg/day scopolamine-treated aged rats	1.5 mg/kg intraperitoneal Se supplementation for 14 days	↓ membrane permeability to Ca^2+^ ↑ membrane phospholipids ↓ reduction of GPx	Se as a neuroprotective factor	[542]
Brain tissue from rats treated with DHA + EPA + uridine (fish oil)	1600 mg/kg vitamin C, 1600 mg/kg E and 1.2 mg/kg Se diet for 6 weeks	↑ membrane phospholipids	Co-supplementation of Se, vitamins, and fish oil promotes synaptogenesis	[543]
Triple transgenic AD mice	6 μg/mL selenomethionine supplementation through drinking water for 12 weeks	↓ extrasynaptic NMDARs activity ↑ synaptic NMDARs activity ↓ membrane permeability to Ca^2+^	Selenomethionine improves synaptic plasticity and cognitive functioning	[544]
Triple transgenic AD mice	6 μg/mL selenomethionine supplementation through drinking water for 12 weeks	↓ total tau and phosphorylated tau ↓ synaptic protein loss	Selenomethionine to restore synapses	[545]
Triple transgenic AD mice	6 μg/mL selenomethionine supplementation through drinking water for 12 weeks	↓ total tau and hyperphosphorylated tau ↓ autophagic dysfunction	Selenomethionine to restore synapses	[546]
Triple transgenic AD mice	6 μg/mL selenomethionine supplementation through drinking water for 12 weeks	↓ tau pathologies	Se supplementation, as a potential tool to improve cognitive deficits related to AD	[547]
Triple transgenic AD mice	12 μg/mL sodium selenate chronic dietary supplementation	↓ tau aggregation	Sodium selenate, as a promising supportive therapy against AD	[548]
iTRAQ proteomics technology in hippocampus of triple transgenic AD mice	9–12 μg sodium selenate per day in drinking water for 4 months	↓ expression of cortical proteins involved in AD pathogenesis	Sodium selenate, as a potential supportive therapeutic agent for AD	[549]
Wistar rats intoxicated with aluminum chloride to mimic AD neurodegeneration	100 mg/kg/day oral resveratrol-Se nanoparticles for 60 days	↑ antioxidant effect compared to Se alone administration	Se as a promising supplementation against AD when combined with resveratrol	[550]
Lymphoblastoid cell lines from AD patients	Resveratrol and Se exposure	↑ antioxidant effect	Se as a protective agent against AD when combined with resveratrol	[551]
APP/Tau/PSEN and APP/PS1 transgenic mouse models	3 or 1.5 μg/g Se and 36 or 18 μg/g folic acid oral co-supplementation	↓ Aβ generation ↓ tau hyperphosphorylation	Se as a potential therapy against AD when combined with folic acid	[552]
AD patients	Combined probiotics (*Lactobacillus acidophilus*, *Bifidobacterium bifidum*, and *Bifidobacterium longum,* 2 × 10^9^ CFU/day each) and 200 mg/day selenium oral supplementation for 12 weeks	↑ antioxidant effect	Se as a potential supportive therapy against AD when combined with probiotics	[553]
Primary cortical and human embryonic kidney cells exposed to Aβ_1–42_	Zn^2+^ incubation	↑ antioxidant effect toward H_2_O_2_ formation	Zn as a neuroprotective factor against AD	[554]
Chinese hamster ovary cells overexpressing amyloid precursor protein	Exposure to Cu or Zn bis (thiosemicarbazonato) therapy	↓ monomeric Aβ peptide	Zn bis (thiosemicarbazonato) as a potential AD supplementation	[555]
Acetylcholinesterase enzymes from Electric eel	Zinc carboxylate derivatives exposure	↓ acetylcholinesterase enzyme activity	Zinc carboxylate derivatives to treat AD	[556]
APP23 mice	Zn nanoparticle injection for 14 days	↓ plaques deposition	↑ brain Zn levels to counteract AD	[557]
Male Sprague Dawley rats receiving aluminum chloride	227 mg/L Zinc sulfate in drinking water for 8 weeks	↓ tau levels ↓ APP levels ↓ α-synuclein levels ↓ alterations in histological architecture	Zn to reverse the effects of aluminum-induced neurodegeneration, which is correlated with AD	[558]
Tg2576 mice treated with Cu	2 g/L Zinc acetate in drinking water for 6 months	↓ ROS formation ↓ amyloid burden ↓ Cu absorption	Zn to reverse the Cu toxic effects, which are correlated with AD	[559]
AD patients	150 mg Zn supplementation for 6 months	↑ Zn levels ↓ Cu levels	Zn therapy to lower Cu absorption and to restore Zn levels, with the aim to protect from cognitive impairment	[560]
Male Wistar rats treated with STZ	Co-administration of 10 mg/kg Zn and 0.1 mg/kg Se intraperitoneally for 1 week	↓ oxidative stress ↓ mitochondrial membranes collapse ↑ GPx ↑ superoxide dismutase	Zn and Se co-administration to improve cognitive functions and prevent the development of AD	[561]
Male Kunming mice	Zn, Se, and fish-oil (EPA + DHA) co-administration for 7 weeks	↓ APP cleavage	Zn, Se and fish-oil co-administration to improve cognitive functions in AD models	[562]
APP2576 transgenic mice	Oral clioquinol (Cu/Zn chelator) administration for 9 weeks	↓ Aβ plaques	Cu/Zn chelators as a supportive therapeutic strategy	[563]
APP/PS1 mouse brain sections	300 μg/mL recombinant human S100A6 protein (Zn chelator) incubation for 12 h or culture with human S100A6-expressing cells	↓ Aβ plaques	Zn sequestration as a supportive therapeutic strategy	[564]
APP/PS1 transgenic mice watered with high quantities of iron	200 mg/kg intranasal deferoxamine (Fe chelator) once every other day for 3 months	↓ Aβ plaques	Deferoxamine as a supplemental treatment for AD	[565]
Traumatic brain injury murine model	Deferoxamine (Fe chelator) intraperitoneal treatment	↓ Aβ plaques ↓ brain ferritin	Deferoxamine as a potential preventive treatment to avoid neurodegeneration in AD patients	[566]
1.14 mg/kg/day scopolamine-treated rats for 7 days	5, 10, 20 mg/kg oral deferiprone (Fe chelator) for 14 days	↓ Aβ plaques	Deferiprone as a potential preventive treatment in AD patients	[567]
rTg(tauP301L)4510 tauopathy murine model	100 mg/kg oral deferiprone (Fe chelator) for 16 weeks	↓ cognitive deficit	Deferiprone as a potential supportive therapy for tauopathies	[568]
Blood samples and brain tissues from NMRI male mice treated with 100 mg/kg/day iron dextran for 4 times a week for 6 weeks	Hesperidin/coumarin/desferal (all Fe chelators) treatment for 4 times a week for 4 weeks	↓ Fe levels ↑ antioxidant enzymatic activity	Hesperidin and coumarin to enhance antioxidant enzymatic activity	[569]
Brain sections from NMRI male mice following treatment with 100 mg/kg/day iron dextran injections for 4 times a week for 4 weeks	30/60 mg/kg/day naringin (Fe chelator) administration for a month	↓ Fe levels ↓ Aβ plaques	Naringin as a preventive supportive treatment for AD	[570]
Brain homogenates from rats	Curcumin, capsaicin, and S-allylcysteine (Fe chelators) exposure	↓ Fe levels ↑ antioxidant effect	Curcumin, capsaicin, and S-allylcysteine as possible tools for the prevention and treatment of AD	[571]
APP/PS1 double transgenic AD mice	M30 (Fe chelator) oral administration 4 times a week for 9 months	↓ Fe levels ↓ APP levels ↓ APP and tau phosphorylation ↓ Aβ plaques	M30 as a potential supportive therapy for AD	[572]
AD murine model under a high-fat diet	0.5 mg/kg M30 oral administration once every 2 days for 1 month	↓ Fe levels ↓ Aβ burden ↓ neuroinflammation ↓ synaptic impairment	High-fat diet as a risk factor for AD and M30 as a potential therapeutic compound	[573]
Human cells and *Caenorhabditis elegans* nematode	20 multifunctional synthetic compounds based on the nicotinoyl hydrazone scaffold, in particular, SNH6 (Fe chelator and NAD^+^ donor)	↓ Fe levels ↓ Aβ burden ↓ oxidative stress ↑ sirtuin	nicotinoyl hydrazone-based compounds, especially SNH6 as a promising supportive therapy for AD	[574]
Human brain micro-vascular endothelial cells	PBT434 (Fe chelator) exposure	↓ Fe reuptake by blood-brain barrier endothelial cells	PBT434, used to prevent Fe-induced cytotoxic effects	[575]
Human plasma	Chelator–nanoparticles systems	↑ blood-brain barrier permeability to Fe chelators	Nanoparticles as a tool to improve chelation treatment for AD	[576]
AD murine model	Fe-enriched water administration for 8 months	↓ Aβ_42_ burden	Fe as a supplemental treatment for AD	[577]
Murine AD model by an ICV injection of Aβ_1–42_ peptide	Oral administration of 25 mg/kg of bis-8-aminoquinoline PA1637 (Cu chelator) three times per week (8 doses in total)	↑ functioning of the episodic memory	PA1637 as a possible supportive treatment for AD	[578]
AD mouse models	Oral administration of 10 mg kg^−1^ TDMQ20 (Cu chelator) in 100 μL of solvent every 2 days for 3 months	↑ cognitive and behavioral performance	TDMQ20 as a possible supplemental treatment for AD	[579]
Mouse brain cells	Flavonoid fisetin (Cu and Fe chelator) exposure	↓ cell death	Fisetin as a neuroprotective compound against AD	[580]
PS19 transgenic murine model	Oral zinc acetate (Cu chelator) treatment	↓ spatial memory deficit in female mice, but not in male ones No significant differences in tau pathology	Cu chelation may improve cognitive symptoms	[581]
HT22 cells	MTDLs (Cu chelator) with a rivastigmine skeleton (inhibitor of AChE)	↓ AChE and BuChE activity ↓ Cu quantities	MTDLs as promising protective compounds for neurons	[582]
In vitro assays	Multifunctional tacrine-7-hydroxycoumarin hybrids chelating Cu and inhibiting AChE and BuChE	↓ AChE and BuChE activity ↓ Cu quantities	Multifunctional agents as promising compounds to treat AD	[583]

Abbreviations: Aβ: *amyloid beta* peptide; AChE: acetylcholinesterase; AD: Alzheimer’s disease; APP: amyloid precursor protein; BuChE: butyrylcholinesterase; CSF: cerebrospinal fluid; Cu: copper; DHA: docosahexaenoic acid; EPA: eicosapentaenoic acid; Fe: iron; GPx: glutathione peroxidase; ICV: intracerebroventricular; iTRAQ: isobaric tags for relative and absolute quantitation; MTDLs: multi-target-directed ligands; NMDAR: N-methyl-D-aspartate acid receptors; NMRI mice: female Naval Medical Research Institute (NMRI) outbred mice; PS1: presenilin 1; ROS: reactive oxygen species; SDAT: sporadic dementia of Alzheimer’s type; Se: selenium; STZ: streptozotocin; Zn: zinc; 3D-ASL: three-dimensional arterial spin labeling; ↑: increase; ↓: decrease.

## Data Availability

Not applicable.
